# Cut the noise or couple up: Coordinating circadian and synthetic clocks

**DOI:** 10.1016/j.isci.2021.103051

**Published:** 2021-08-27

**Authors:** Chris N. Micklem, James C.W. Locke

**Affiliations:** 1 The Sainsbury Laboratory, University of Cambridge, Bateman Street, Cambridge CB2 1LR, UK; 2 The Cavendish Laboratory, Department of Physics, University of Cambridge, JJ Thomson Avenue, Cambridge CH3 0HE, UK

**Keywords:** Biological sciences, Physiology, Developmental biology, Chronobiology, Biophysics, Mathematical biosciences

## Abstract

Circadian clocks are important to much of life on Earth and are of inherent interest to humanity, implicated in fields ranging from agriculture and ecology to developmental biology and medicine. New techniques show that it is not simply the presence of clocks, but coordination between them that is critical for complex physiological processes across the kingdoms of life. Recent years have also seen impressive advances in synthetic biology to the point where parallels can be drawn between synthetic biological and circadian oscillators. This review will emphasize theoretical and experimental studies that have revealed a fascinating dichotomy of coupling and heterogeneity among circadian clocks. We will also consolidate the fields of chronobiology and synthetic biology, discussing key design principles of their respective oscillators.

## Graphical abstract

**Figure:**
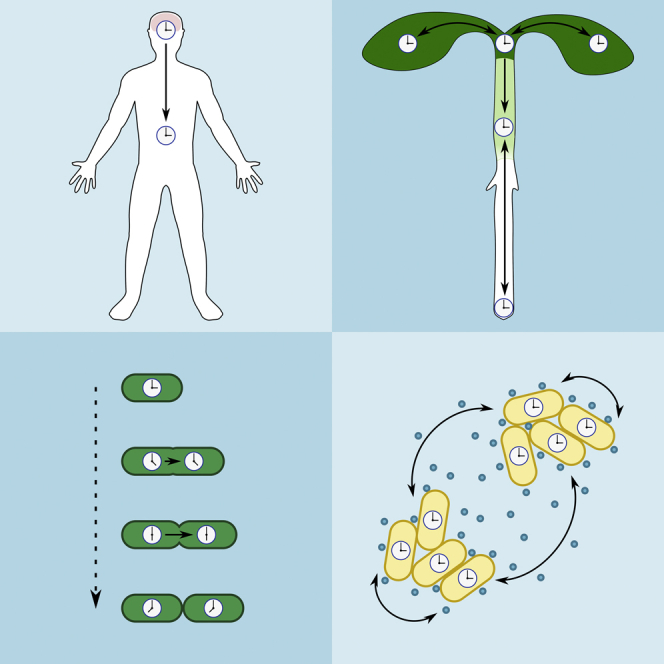

## Introduction

Life on Earth has evolved under the influence of regular, planetary rotation-induced oscillations in environmental conditions. These oscillations, which have slowed over the eons to a familiar ∼24-hr period, have led much of known life to develop mechanisms for timing cellular processes in relation to these changes: circadian clocks. Circadian clocks allow organisms to predict the regular day-night cycles of the planet; undergoing physiological changes in anticipation of these and, in doing so, conferring fitness advantages. Present-day circadian clocks possess defining features including growth rate-independent (i.e., clock speed unaffected by growth rate), temperature-compensated (i.e., clock speed unaffected by temperature), entrainable (i.e., can synchronize to external signals) and self-sustained (i.e., continues in the absence of entraining signals) oscillations with a ∼24-hr period. They anticipate daily changes to optimally time key life processes, such as metabolism (Beck et al., 2001; Rascher et al., 2001; Bray et al., 2008), DNA repair (Gaddameedhi et al., 2011), and regeneration (Karpowicz et al., 2013; Stokes et al., 2017). The advantages circadian clocks confer to organisms under Earth’s diel rhythms are so fundamental they likely convergently evolved at least twice, appearing in various forms across the Tree of Life (Rosbash, 2009).

However, while the importance of circadian clocks is clear, there is much yet to learn about their workings. A multitude of methods, ranging from molecular genetics to single-cell imaging, have been instrumental in revealing circadian clocks to be single-celled oscillators, occurring in virtually every cell of clock-possessing organisms. As techniques advance, it has been shown that coupling (i.e., ability of clocks to synchronize to each other) between these oscillators can be critical for proper circadian coordination, at both local and whole-organism scales. Conversely, it also appears that in some systems, coupling is weak or absent, increasing potential for intercellular circadian heterogeneity (i.e., variation in timing between individual clocks). Such insight has been obtained through pairing modern experimental methods with mathematical modeling approaches, allowing both direct observation of single-cell circadian dynamics and inference of single-cell information from population-level observations. These systems biological approaches have allowed for reverse-engineering of existing circadian oscillators, while synthetic biological forward-engineering has resulted in the creation of impressive synthetic oscillators.

Here, following brief introductions to the current mechanistic understanding of mammalian, plant, and cyanobacterial clocks, we will review experimental and theoretical evidence for coupling, or lack thereof, among single-cell circadian oscillators across the kingdoms of life. Parallels will also be drawn with synthetic biological oscillators as they converge upon their naturally evolved counterparts, as we explore design principles and network structures core to their function.

Before exploring evidence for coupling in different circadian systems, it will be useful to understand the basic molecular clockwork of three main clock models: animals, plants, and cyanobacteria. First, we will visit animal clocks. While several orthologous animal clock models are used, notably including *Drosophila melanogaster*, in which many foundational chronobiological genetic experiments were conducted (Konopka and Benzer, 1971; Bargiello et al., 1984; Zehring et al., 1984), we will place specific focus upon mammals, where most study of animal clock coupling has taken place.

## The circadian clock in mammals

**Figure 1:**
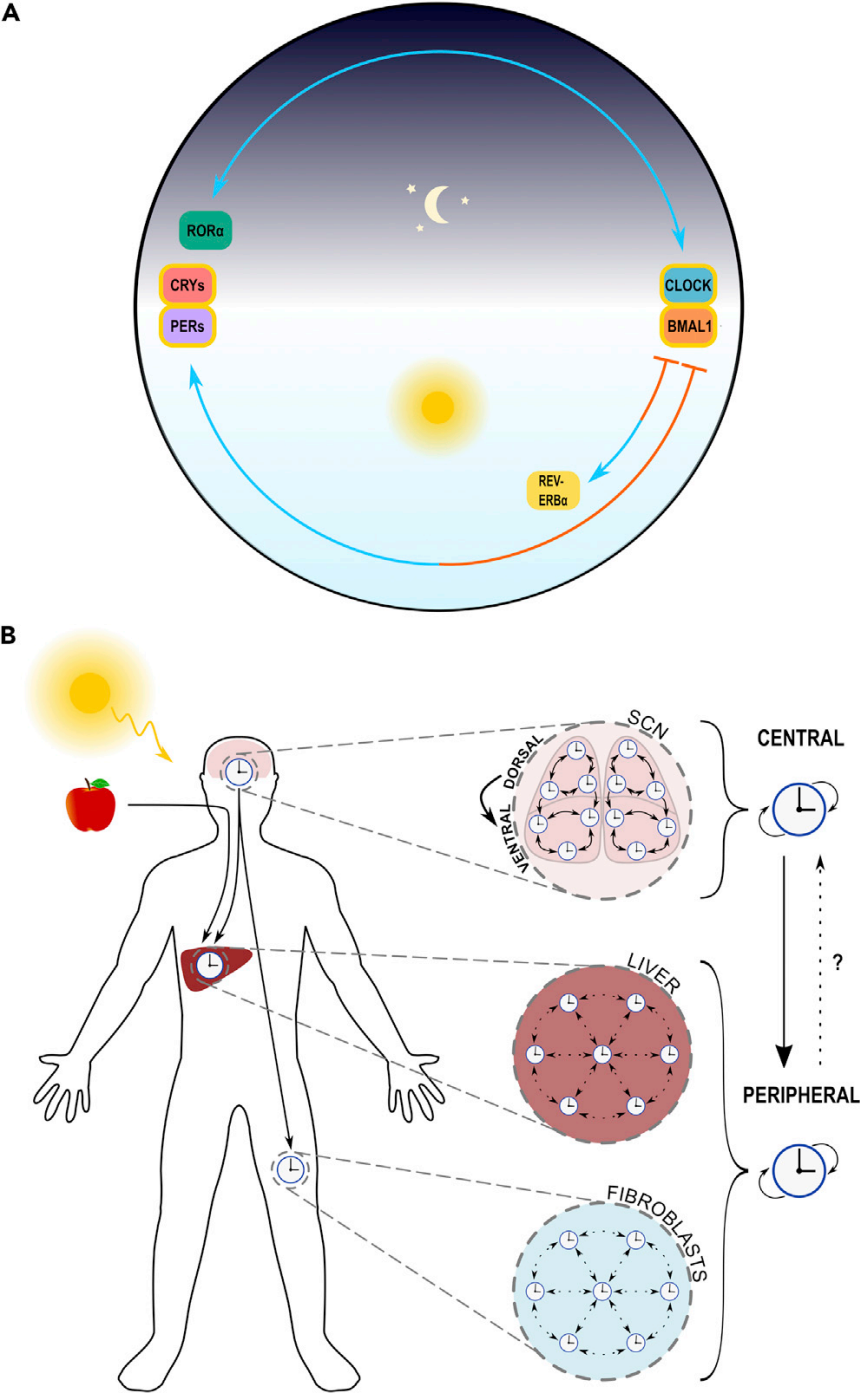
The circadian clock in mammals (A) Simplified molecular clock mechanism. Transcription factors CLOCK and BMAL1 heterodimerize, activating E-box enhancer-containing genes, notably *Per*s and *Cry*s. PER and CRY proteins also heterodimerize, transrepressing themselves by negatively feeding back upon the CLOCK:BMAL1 complex (Gekakis et al., 1998; Kume et al., 1999; Shearman et al., 2000). The CLOCK:BMAL1 heterodimer also activates E-boxes of nuclear receptor genes *Rev-erbα* and *Rorα* (Triqueneaux et al., 2004). REV-ERB and ROR compete to bind RREs in the *Bmal1* promoter, repressing and activating *Bmal1*, respectively (Preitner et al., 2002; Akashi and Takumi, 2005; Guillaumond et al., 2005). Components are positioned on a 24-hr dial (top is midnight) corresponding to approximate peak expression time. Gold borders indicate protein complexes. Positive and negative regulation signified by blue pointed arrows and orange flat-headed arrows, respectively. Where regulation is bidirectional, double-ended arrows are used. (B) Circadian organization. Three key clocks studied: SCN, liver, and fibroblast are shown in the context of a human body, along with two main zeitgebers: sunlight and food. Strongly coupled SCN clocks are entrained by light and coordinate generally weakly coupled peripheral clocks in a centralized hierarchy. Liver clocks are also subject to entrainment via food. Though circadian influence appears largely unidirectional from central to peripheral oscillators, some evidence exists for peripheral feedback on central oscillators (Myung et al., 2018). Arrows represent entraining influence within the circadian hierarchy and coupling between cellular clocks: solid lines indicate strong influence, dotted lines indicate weak influence.

The mammalian circadian clock is built up of transcriptional-translational feedback loops (TTFLs) generating ∼24-hr oscillations in clock gene expression and subsequently in cellular and organism-level behavior. Although these single-cell clocks are arranged hierarchically whereby clocks of the suprachiasmatic nucleus (SCN) in the mammalian brain coordinate clocks of peripheral tissues, their core circadian oscillators remain the same. While the gene regulatory network of the mammalian circadian clock is highly complex, it has traditionally been distilled down to TTFLs comprising *Clock*, *Bmal1*, *Pers*, *Crys*, *Rev-erbα*, *Rorα*, their products and regulatory targets (Figure 1A).

These TTFLs interact, generating ∼24-hr rhythms in expression and, through direct and indirect action upon ROR/REV-ERB-response element (RRE), E-box and D-box-containing regulatory sequences, coordinate timing of clock-controlled gene (CCG) expression (Ueda et al., 2005; Ukai-Tadenuma et al., 2011; Yoshitane et al., 2019). CCGs are in turn involved in a multitude of processes including metabolism (Bray et al., 2008), insulin secretion (Marcheva et al., 2010), DNA repair (Gaddameedhi et al., 2011) and regeneration (Karpowicz et al., 2013; Stokes et al., 2017). Fluorescent and bioluminescent reporters allow mammalian CCGs and clock genes to be observedin vitro or even in vivo. These have been instrumental in studying individual cellular clocks and their interactions. Additionally, mathematical modeling has been indispensable in characterizing behaviors of oscillator populations under different coupling regimes. As such, we will focus on both experimental and theoretical studies, highlighting how these complementary approaches help elucidate intercellular clock coupling.

## Mammalian clocks show varied coupling in central and peripheral organs

In this section, we will focus largely on *intraorgan* cell-to-cell clock coupling and will not cover all the many facets of mammalian clock coupling in comprehensive detail, as this has already been rigorously reviewed (Pilorz et al., 2020; Koronowski and Sassone-Corsi, 2021).

Mammalian circadian clocks are traditionally viewed as a hierarchical system of central oscillators, located in the SCN of the mammalian brain’s anterior hypothalamus, coordinating peripheral oscillators throughout the rest of the body (Figure 1B). The SCN is the conduit through which other clocks can be entrained by light, via signals transduced by intrinsically photosensitive retinal ganglion cells of the retinohypothalamic tract (Berson et al., 2002; Hattar et al., 2002). Light is the main entraining cue, or zeitgeber, for circadian mechanisms across the kingdoms of life. When all zeitgebers are removed, circadian oscillators transition to a free-run. Fundamental oscillator properties, including their coupling, can be understood by observing dynamics in this free-running state. The importance of the SCN in driving rhythms in the mammalian circadian hierarchy has been evident from many ingenious lesion, transplantation, and mutant studies conducted over the past half-century (Stephan and Zucker, 1972; Ralph and Menaker, 1988; Ralph et al., 1990; Sakamoto et al., 1998; Pando et al., 2002). Early single-cell observation of SCN neuron firing rates on fixed microelectrode arrays (Inouye and Kawamura, 1979) also revealed neurons to contain individual self-sustained oscillators. However, results were initially conflicted as to the absence (Welsh et al., 1995) or presence (Honma et al., 1998) of coupling.

**Figure 2:**
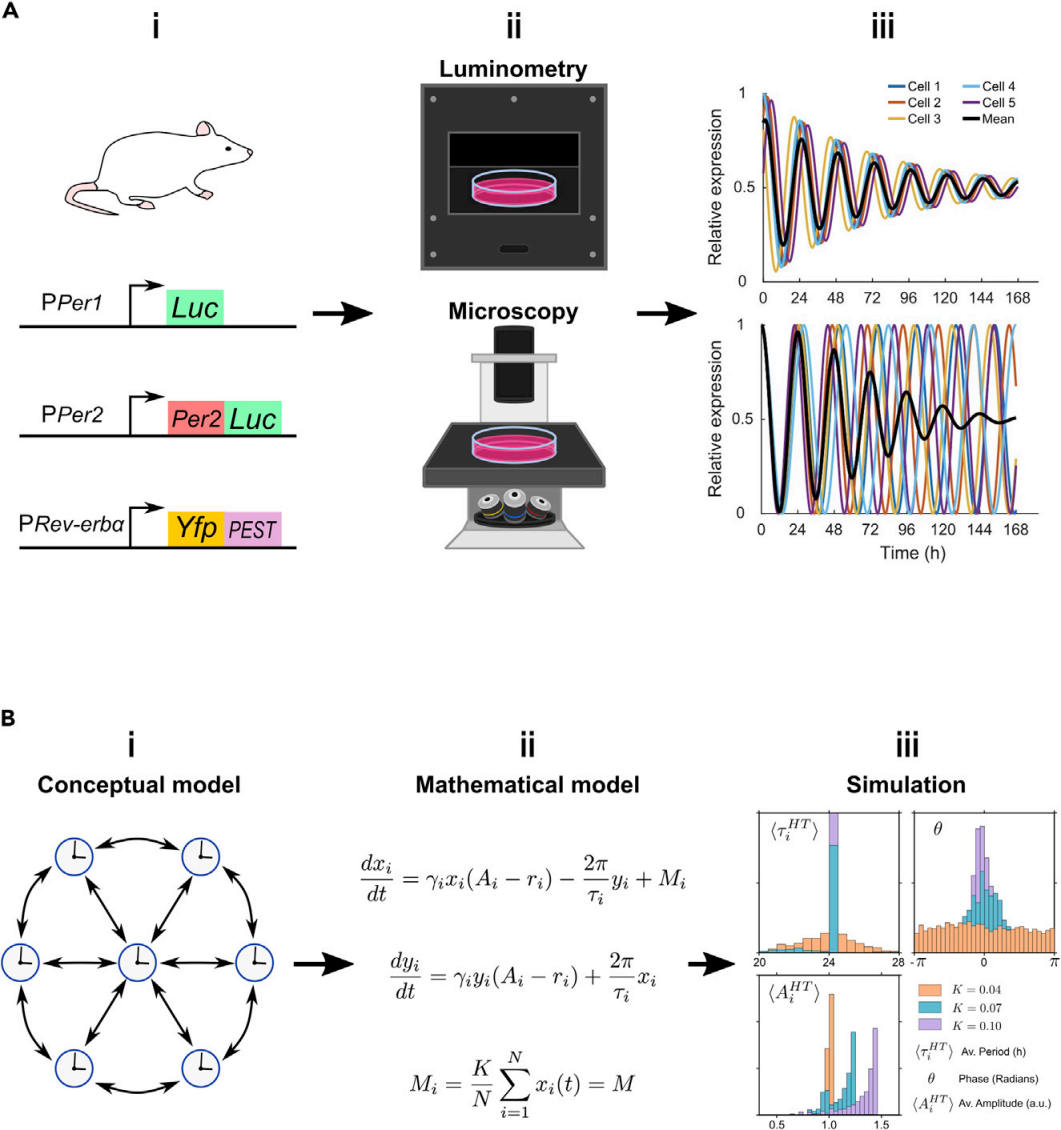
Studying coupling in mammals (A) Key experimental techniques. (i) Rodents are predominant models for studying coupling in mammals. A variety of bioluminescent and fluorescent transcriptional and translational clock gene reporters are used (examples shown). Fluorescent reporters feature degradation tags (e.g., PEST) to prevent buildup. (ii) These can be observed using time-lapse population-level luminometry (bioluminescent reporters) or population- and single-cell-level microscopy (bioluminescent and fluorescent reporters) of tissue explants and dissociated cells, under constant zeitgeber conditions. (iii) Though population- and single-cell-level observations are useful, the latter facilitates better understanding of oscillator dynamics, e.g., population-level damping (black lines) could be due to damping (top) or phase dispersion (bottom) of individual cellular oscillators (colored lines) (figure adapted, under Creative Commons Attribution Licence version 4.0 (CC BY 4.0), from Gould, P. D. et al. (2018) ‘Coordination of robust single cell rhythms in the Arabidopsis circadian clock via spatial waves of gene expression’, eLife, 7. https://doi.org/10.7554/eLife.31700. Copyright 2018 by Gould et al.). (B) Key theoretical techniques (representing approach from Schmal et al. (2018)). (i) Modeling clock coupling starts with a conceptual model of how clocks interact. (ii**)** This can be represented as differential equations for mean-field coupled phase-amplitude *Poincaré* oscillators. (iii**)** Simulations can characterize expected period, phase, and amplitude distributions under different coupling regimes (varying coupling strength, *K*): purple is strong (*K* = 0.1), blue is intermediate (*K* = 0.07), orange is weak (*K* = 0.04). Stronger coupling results in narrower period, phase distributions, and amplitude expansion, while lowering coupling has the opposite effect (figure redrawn and adapted, with permission, from Schmal, C., Herzog, E. D. and Herzel, H. (2018) ‘Measuring Relative Coupling Strength in Circadian Systems’, Journal of biological rhythms, 33(1), pp. 84–98. https://doi.org/10.1177/0748730417740467. Copyright 2018 by SAGE Publishing).

**Figure 3:**
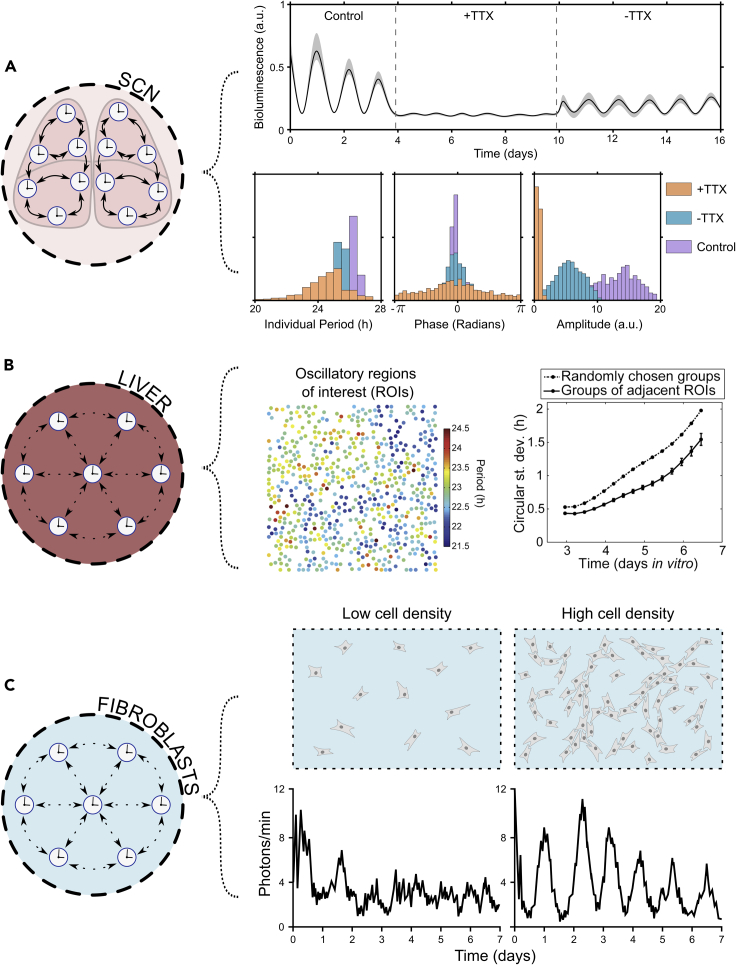
Evidence for coupling in mammals (A) Coupling in the SCN (representing results from Schmal et al. (2018)). The strongest evidence for coupling in the SCN has been demonstrated most clearly through blocking coupling with tetrodotoxin (TTX) to abrogate synchronous oscillations, followed by a period of resynchronization after washing it out, to obtain high (pre-TTX, purple), low (+TTX, orange), and intermediate (-TTX wash, blue) coupling regimes. Black line is the mean and gray area represents the standard deviation in PER2::LUC bioluminescence of single SCN neurons. Vertical dashed lines indicate time of TTX addition (day 4) and washing out (day 10). Observed changes in period, phase, and amplitude distributions are impressively concordant with predictions from mathematical modeling (Figure 2B): Strong pre-TTX coupling (purple) results in narrow period, phase distributions and high amplitudes; weak +TTX coupling (orange) results in phase, period dispersion and low amplitudes; intermediate -TTX coupling (blue) results in narrower, intermediate phase, period distributions and amplitude expansion. These data indicate coupling and suggest a role of electrochemical signaling in its mediation (figure redrawn and adapted, with permission, from Schmal, C., Herzog, E. D. and Herzel, H. (2018) ‘Measuring Relative Coupling Strength in Circadian Systems’, *Journal of biological rhythms*, 33(1), pp. 84–98. https://doi.org/10.1177/0748730417740467. Copyright 2018 by SAGE Publishing). (B) Coupling in the liver (results from Guenthner et al. (2014)). (i) Oscillatory regions of interest (ROIs), taken to be individual PER2::LUC hepatocytes, show spatial structure in their periods (color coded) (figure adapted, under Creative Commons Attribution license version 4.0 (CC BY 4.0), from Guenthner, C. J. et al. (2014) ‘Circadian rhythms of PER2::LUC in individual primary mouse hepatocytes and cultures’, *PLoSOne*, 9(2), p. e87573. https://doi.org/10.1371/journal.pone.0087573. Copyright 2014 by Guenthner et al.). (ii) Groups of 7 adjacent ROIs had significantly lower circular standard deviation than groups of 7 randomly chosen ROIs over a 1-week period (solid circles are the mean and error bars represent the standard error). These data indicate nearby cells are closer in phase to each other than distant cells, consistent with coupling (figure adapted, under Creative Commons Attribution license version 4.0 (CC BY 4.0), from Guenthner, C. J. et al. (2014) ‘Circadian rhythms of PER2::LUC in individual primary mouse hepatocytes and cultures’, *PLoSOne*, 9(2), p. e87573. https://doi.org/10.1371/journal.pone.0087573. Copyright 2014 by Guenthner et al.). (C) Coupling among fibroblasts (representing results from Noguchi et al. (2013)). Fibroblast coupling is most apparent from results demonstrating a clear density dependency in clock gene rhythmicity in PER2::LUC fibroblasts: representative single-cell traces show (i) cells lose rhythmicity at low density (ii**)** but regain it at high density (figures redrawn and adapted, with permission, from Noguchi, T., Wang, L. L. and Welsh, D. K. (2013) ‘Fibroblast PER2 circadian rhythmicity depends on cell density’, *Journal of biological rhythms*, 28(3), pp. 183–192. https://doi.org/10.1177/0748730413487494. Copyright 2013 by SAGE Publishing).

Development of bioluminescent and fluorescent reporter lines has greatly facilitated real-time observation of clocks (Figure 2A). These are comparatively noninvasive, while allowing longitudinal study with high spatial resolution. Bioluminescent transcriptional (Yamaguchi et al., 2000, 2003; Yamazaki et al., 2000, 2002) and translational (Yoo et al., 2004) clock reporter mouse lines have revealed spatiotemporal waves of clock gene expression across the SCN. Furthermore, free-running SCN neurons exhibit remarkable ability to maintain coherent oscillations for >50 days, indicative of coupled, self-sustained oscillators. Complementary observation of decreasing neuronal rhythmicity and synchrony when Na+ channels, required for generating action potentials, are blocked with tetrodotoxin (TTX) (Yamaguchi et al., 2003; Webb et al., 2009; Abel et al., 2016; Taylor et al., 2017) as well as when grown at low densities (Webb et al., 2009) has further highlighted the importance of electrochemical signal-mediated coupling in maintaining rhythmicity and cell-to-cell coordination (Figure 3A). Overall, coupling between SCN subcompartments and neurons is thought to be mediated by a combination of paracrine (Maywood et al., 2011), synaptic (via *γ*-aminobutyric acid (GABA), vasoactive intestinal polypeptide (VIP), arginine vasopressin (AVP), gastrin-releasing peptide (GRP) (Albus et al., 2005; Maywood et al., 2006) and cytoplasmic signaling (via cyclic adenosine monophosphate (cAMP) and Ca^2+^ ions) (Lundkvist et al., 2005; O’Neill et al., 2008).

Given the significance of the SCN within the mammalian circadian hierarchy and experimental evidence for coupling within the SCN, one might wonder what of peripheral clocks? Peripheral clocks previously gained attention when immortalized fibroblast cells, long-absent of SCN signaling, exhibited serum shock-coordinated circadian gene expression. This implied possible independence from the SCN (Balsalobre et al., 1998; Nagoshi et al., 2004; Welsh et al., 2004). Furthermore, like the SCN, peripheral clocks of the liver, lung, and dissociated fibroblasts sustain circadian oscillations for up to 20 days, with hepatocytes showing proximity-phase relationships, indicative of coupling (Figure 3B) (Yoo et al., 2004; Liu et al., 2007; Guenthner et al., 2014). Lesioning of the SCN to disrupt potential centralized maintenance of peripheral clocks does not abolish oscillations in these peripheral tissues, further alluding to self-sustained and coupled peripheral clocks (Yoo et al., 2004). Moreover, akin to the density-dependent rhythmicity of SCN neurons (Webb et al., 2009), density-dependency has also been described in fibroblast clocks (Figure 3C), consistent with coupling (Noguchi et al., 2013). Low-density bioluminescent reporter cells have rhythmicity rescued when cultured with high-density non-luminescent wild type and nonrhythmic *Bmal1-/-* cells as well as with conditioned media from high-density cultures, suggesting a nonrhythmic, paracrine coupling signal maintains rhythmicity (Noguchi et al., 2013). This holds true when co-cultured with long-period mutant fibroblasts, indicating that although fibroblast coupling might maintain rhythmicity, it does not appear to influence cell-to-cell synchronization (Noguchi et al., 2013).

Although peripheral clocks may possess coupling, it is not necessarily of comparable strength to that within the SCN. Indeed, while SCN coupling is reportedly capable of maintaining population-level rhythmicity in otherwise arrhythmic *Per1-/-* and *Cry1-/-* mutant cells, this is not observed for lung, liver, and cornea explants (Liu et al., 2007), highlighting possible fundamental differences in the coupling capability of peripheral tissues.

Real-time, longitudinal bioluminescent circadian reporter studies in free-moving mice have helped further unveil the nature of peripheral coupling (Tahara et al., 2012; Saini et al., 2013). These focus particularly on liver clocks, which are known to synchronize to feeding rhythms (Damiola et al., 2000; Stokkan et al., 2001). Interestingly, entrainment to feeding rhythms occurs more easily in SCN-lesioned mice, suggesting separate, competing entrainment pathways are at work (Tahara et al., 2012; Saini et al., 2013). Later study examining mRNA levels in clock-mutant mice with clocks reconstituted only in the liver concluded that coherent liver clock rhythms nonetheless rely upon systemic signals from other clocks and an SCN-mediated, yet apparently clock-independent transduction of rhythmic light cues (Koronowski et al., 2019). However, most recently, by combining the real-time recording techniques of Saini et al. (2013) with liver-specific clock reconstitution akin to Koronowski et al. (2019), it has been shown that hepatocytes do indeed maintain a degree of phase coherence even in the absence of the SCN and other non-hepatic clocks (Sinturel et al., 2021). One explanation for the inconsistency between the Koronowski et al. (2019) and Sinturel et al. (2021) studies could be the differing methods used to assess rhythms: qPCR in the former and real-time bioluminescence recording in the latter. While qPCR can be a highly effective tool for studying circadian rhythms, if mice harvested at each time point are not in the same phase, this can result in an apparent lack of rhythms. Real-time recording, however, permits tracking of each mouse at all time points, facilitating the identification of true rhythms. This is especially useful in the context of these studies, in which mice that are behaviorally arrhythmic under constant conditions are observed. In such studies, where phase coherence between individuals cannot be easily ascertained, real-time recording can be a particularly powerful technique.

While experimental evidence for long-term and density-dependent oscillations in both the SCN and peripheral tissues might intuitively indicate coupled, self-sustained oscillators, mathematical modeling allows this to be better resolved. Theoretical study of generic oscillator representations has been translated with great success to describe circadian clocks (Goodwin, 1965; Winfree, 1967; Kuramoto, 1975, 1984; Rössler, 1976; Ermentrout and Kopell, 1991; Kiss et al., 2002). Representing clocks as coupled *phase oscillators* (i.e., self-sustained oscillators represented by a single phase variable) as in the celebrated Kuramoto model, or coupled weakly nonlinear oscillators (i.e., approximated to be harmonic, e.g., sinusoidal oscillators) such as classic *van der Pol* oscillators, have been particularly noteworthy in allowing deduction of fundamental clock-coupling properties, even prior to the advent of real-time clock reporters and elucidation of molecular mechanisms.

For example, describing individual SCN clocks as coupled, weakly nonlinear oscillators with stable limit cycles (i.e., self-sustained oscillations) can explain the coordinated outputs from otherwise phase-dispersed neurons (Liu et al., 1997; Achermann and Kunz, 1999). Such models have, importantly, been able to account for stochastic effects and produce experimentally testable predictions for photoentraining coupled clock populations (Achermann and Kunz, 1999; Kunz and Achermann, 2003). It should be noted, however, that modeling mammalian clocks as damped, noise-sustained oscillators has also been shown to effectively fit the single-cell data (Westermark et al., 2009).

Incorporating molecular mechanistic information into models has also been used to recreate clock behaviors. This can be achieved through representing individual clocks as *Goodwin* oscillators, which capture oscillatory dynamics of clock genes through differential equations describing their synthesis and feedback repression (Goodwin, 1965). Further introduction of coupling via a mean-field, whereby the influence of all oscillators on any given oscillator is approximated to a single average value, has proven powerful for studying coupled circadian networks (Gonze et al., 2005; Bernard et al., 2007; Kim et al., 2014). Additionally, efforts have been made at complex clock-coupling models, recapitulating clock behavior from more complete clock networks (Bernard et al., 2007; Hafner et al., 2012).

The spatiotemporal patterns observable through real-time clock reporters can also reveal much about the underlying coupling in clock populations. Notably, Moran’s *I*, describing the *spatial autocorrelation* in phase coherence (i.e., the degree to which oscillators are synchronized in relation to their proximity) has been successfully applied to circadian systems (Schmal et al., 2017). It was recently used to reveal statistically significant spatiotemporal patterns formed by the peripheral clocks of the choroid plexus (CP) (Myung et al., 2018). These were comparable to patterns produced by a nearest-neighbour-coupled phase-amplitude *Poincaré* oscillator model (Glass and Mackey, 1988), suggesting neighboring CP cells may be coupled directly via gap junctions (Myung et al., 2018). This was experimentally supported by pharmacological and physical perturbation of gap junctions, which exhibited striking dose-dependent CP clock damping (Myung et al., 2018). Surprisingly, this coupling appeared to confer more robust and synchronous oscillations than even the SCN, with CP clocks potentially influencing SCN clocks through the cerebrospinal fluid (Myung et al., 2018). Thus, while the SCN may be the main circadian coordinator, this does not necessarily mean it has the strongest coupling, nor that influence between central and peripheral clocks is strictly unidirectional.

The challenge of quantifying relative coupling strength in different systems can also be aided by theoretical study. By applying assertions of the widely used Kuramoto model (Kuramoto, 1975, 1984; Strogatz, 2000) to corroborate predictions from oscillator theory with real chronobiological data, this has culminated recently in formalisms, describing characteristics of circadian oscillator systems under different coupling regimes (Schmal et al., 2018). By observing the distributions of three features: period, phase, and amplitude (Figure 2B), the two main variants of coupling observed in circadian systems, undercritical (found in incoherent circadian systems) and overcritical coupling (found in coherent, fully synchronized circadian systems), can then be identified. In undercritical coupling regimes, when coupling is increased, period distributions narrow and frequency-locked clusters emerge. In overcritical coupling regimes, when coupling is increased, phase distributions narrow as the population forms a giant frequency-locked cluster. Finally, increasing coupling also leads to amplitude expansion from synchronization-induced resonance effects. Significantly, this modeling approach fits experimental data for SCN neurons under varying TTX-perturbation regimes (Figure 3A), allowing determination of its effect on relative coupling strength (Abel et al., 2016). Studies describe similar changes in period, phase, and amplitude in density and temperature-perturbed SCN and fibroblast clocks (Webb et al., 2009; Noguchi et al., 2013; Abraham et al., 2018), highlighting links between growth conditions and coupling in different cell types.

Most recently, researchers have developed methods for reducing the high dimensionality of the Kuramoto model with large oscillator numbers (Ott and Antonsen, 2008; Hannay et al., 2018). In particular, application of these has helped to explain counterintuitive phase-response phenomena in different SCN subcompartments, subsequently making interesting predictions on seasonal changes to coupling strength (Hannay et al., 2020).

Through experimental and theoretical study, it appears that most, if not all cells in the body contain a seemingly self-sustained circadian clock, for which coupling helps maintain rhythmicity (Noguchi et al., 2013) and SCN signals are the main synchronizer (Pando et al., 2002; Welsh et al., 2004; Rougemont and Naef, 2007; Koronowski et al., 2019). The degree of reliance upon SCN signals, either direct or indirect, as well as degrees of intrinsic heterogeneity do appear to vary between tissues, however. In fact, this inter-oscillator heterogeneity may highlight features important to mammalian clock functioning, as we will explore next.

## Varied coupling and intrinsic noise in mammalian clocks manifests as heterogeneity

Despite widespread coupling in mammals, peripheral clocks nonetheless exhibit a degree of heterogeneity and discoordination without SCN signals. Understanding such heterogeneity is of interest, as the stochasticity from which it can emerge is increasingly implicated in the functioning of mammalian clocks (Leise et al., 2012) and also in discoordinated states associated with aging (Nakamura et al., 2015) and disease (Li et al., 2013; Kolbe et al., 2019).

This heterogeneity is perhaps best described in fibroblasts, which display self-sustained oscillations when both immortalized or recently isolated, yet exhibit population-level damping, associated with intercellular desynchrony (Figure 2Aiii) (Nagoshi et al., 2004; Welsh et al., 2004). Modeling fibroblasts with weak all-to-all coupling, where they all influence each other with paracrine signals, best explains this (Rougemont and Naef, 2007). Damping is proposed to occur due to this paracrine coupling being subthreshold: insufficient to overcome intrinsic noise (i.e., stochastic gene expression) and maintain synchrony. But why would fibroblasts exchange only subthreshold signals, giving rise to heterogeneity? It is postulated that this increases responsiveness to SCN zeitgebers (Rougemont and Naef, 2007). However, it is also possible that paracrine or other coupling simply *appears* subthreshold due to interference from experiment setups. Indeed, this is a caveat of all such studies. For example, though coupling between SCN neurons is now accepted, early studies had concluded they were uncoupled, likely due to overdispersed culture conditions (Welsh et al., 1995).

Recent extension of previous mammalian clock models (Zhang et al., 2009; Hirota et al., 2012; Leise et al., 2012; St John et al., 2014) by St John and Doyle III, has shed more light upon the stochastic noise underlying fibroblast clocks (St John and Doyle, 2015). Utilizing genome-wide siRNA knockdown and small molecule perturbation data, St John and Doyle III simulated effects of increasing noise in mammalian clock TTFLs. This reliably modeled dose-dependent increases in population-level damping rate, attributable to small molecule- and genetic perturbation-induced increases in single-cell oscillator noise. Importantly, although this study did not quantify fibroblast coupling strength, it nevertheless further demonstrated that the intrinsic noise of fibroblast clocks could sufficiently explain population-level damping over time.

Regardless of true physiological coupling strength, quantifying heterogeneity among fibroblasts, by collecting single-cell data over long periods, has helped reveal advantageous oscillator properties. Collection of such data and application of novel statistical metrics by Leise et al. in 2012 has revealed significant heterogeneity in fibroblast clock period and rhythmicity, owing to intrinsic noise (Leise et al., 2012). On fitting a stochastic model comprising a modified *Goodwin* oscillator (Goodwin, 1965), making use of the Gillespie algorithm (Gillespie, 1977), these data are consistent with fibroblast clocks operating at a period variability optimum, just above a Hopf bifurcation (i.e., where oscillations arise), implying they are self-sustained oscillators. However, the natural parameter distribution of these clocks likely spans below the Hopf bifurcation, which, in a deterministic system, would lead to loss of oscillations. Yet, in a more representative stochastic system, noise may in fact sustain fibroblast clocks at this optimal point, enhancing oscillations and facilitating entrainment (Leise et al., 2012). Thus, while this intrinsic noise might present as heterogeneity, it may also aid in the proper functioning of these clocks.

The latest high-throughput data collection and analysis pipelines have greatly facilitated the study of mammalian clock heterogeneity. Most recently, long-term single-cell and clonal-population bioluminescence tracking has allowed investigations into its heritability (Nikhil et al., 2020; Li et al., 2020a, 2020b). Li et al. (2020c) observing hundreds of heterogeneous mouse ear fibroblast cells over time, establishing 150 clonal cell lines, then tracking single cells and subsequent subcloned populations from these. Concurrently, Nikhil et al. tracked the period distributions of clonal *U-2 OS* human osteosarcoma-derived cell lines, sequentially selected from the tails of parental distributions. While Nikhil et al. observed a period divergence in short- and long-period clonal cell lines, suggesting a heritable component to clock heterogeneity (Nikhil et al., 2020), Li et al. did not find such a divergence from looking at single cells (Li et al., 2020a). Nevertheless, both studies agreed that non-genetically heritable factors were the primary driver of circadian heterogeneity in these systems, particularly thought to drive longer periods (Nikhil et al., 2020; Li et al., 2020a). Perturbation experiments further ratified this, whereby idoxuridine-induced transcriptional noise resulted in significantly increased circadian period, variance, and population-level damping relative to DMSO controls (Li et al., 2020a). Further investigation also identified some epigenetic heritability in fibroblast clock (Li et al., 2020b) and *U-2 OS* clock heterogeneity (Nikhil et al., 2020). This was apparent from knockdown of DNA methyltransferases, responsible for epigenetically heritable heterogeneity in fibroblasts, producing comparable effects to idoxuridine treatment (Li et al., 2020b). Similarly, in *U-2 OS* cells, higher upstream CpG island methylation, specifically in the gene *BHLHE40*, was found in shorter circadian periods (Nikhil et al., 2020). Interestingly, DNA methylation has been associated with suppressed transcriptional noise (Huh et al., 2013) and may explain why periods increase when methylation is decreased. In addition, the study from Nikhil et al. further explored the role of epigenetic modifications in the form of histone acetylation. Upregulating expression with a deacetylase inhibitor intriguingly had no effect on short-period clones and, perhaps unexpectedly, significantly decreased the period of long-period clones, suggesting some epigenetically heritability factors may even suppress noise and heterogeneity (Nikhil et al., 2020). While the exact mechanisms by which these phenomena occur are not yet clear, these results broadly substantiate predictions from mathematical modeling that intrinsic noise drives heterogeneity, manifested as damping, among peripheral clocks (Hirota et al., 2012; Leise et al., 2012; St John et al., 2014; St John and Doyle, 2015).

SCN neurons are similarly affected by noise, evidenced by individual neurons possessing much higher cycle-to-cycle period variability than intact SCN explants or whole-organism behavior (Herzog et al., 2004). Furthermore, mathematical modelling-complemented experiments have demonstrated that intrinsic noise can induce remarkable oscillations, even in *Bmal1* mutants, when SCN neurons are coupled (Ko et al., 2010). Indeed, though intrinsic heterogeneity has been modeled to actively disrupt synchronization in strongly coupled circadian systems, it conversely seems required for achieving coordination when coupling is weaker (Gu et al., 2015, 2016). Better understanding of circadian heterogeneity may therefore help reveal the true nature of coupling in these systems.

## The circadian clock in plants

The circadian clock of plants, like other eukaryotes, comprises intertwined TTFLs which interact, generating ∼24-hr rhythms. The core genes involved do not appear to be orthologous to those of animals, however, raising the possibility of multiple eukaryotic clock origins (Rosbash, 2009).

**Figure 4:**
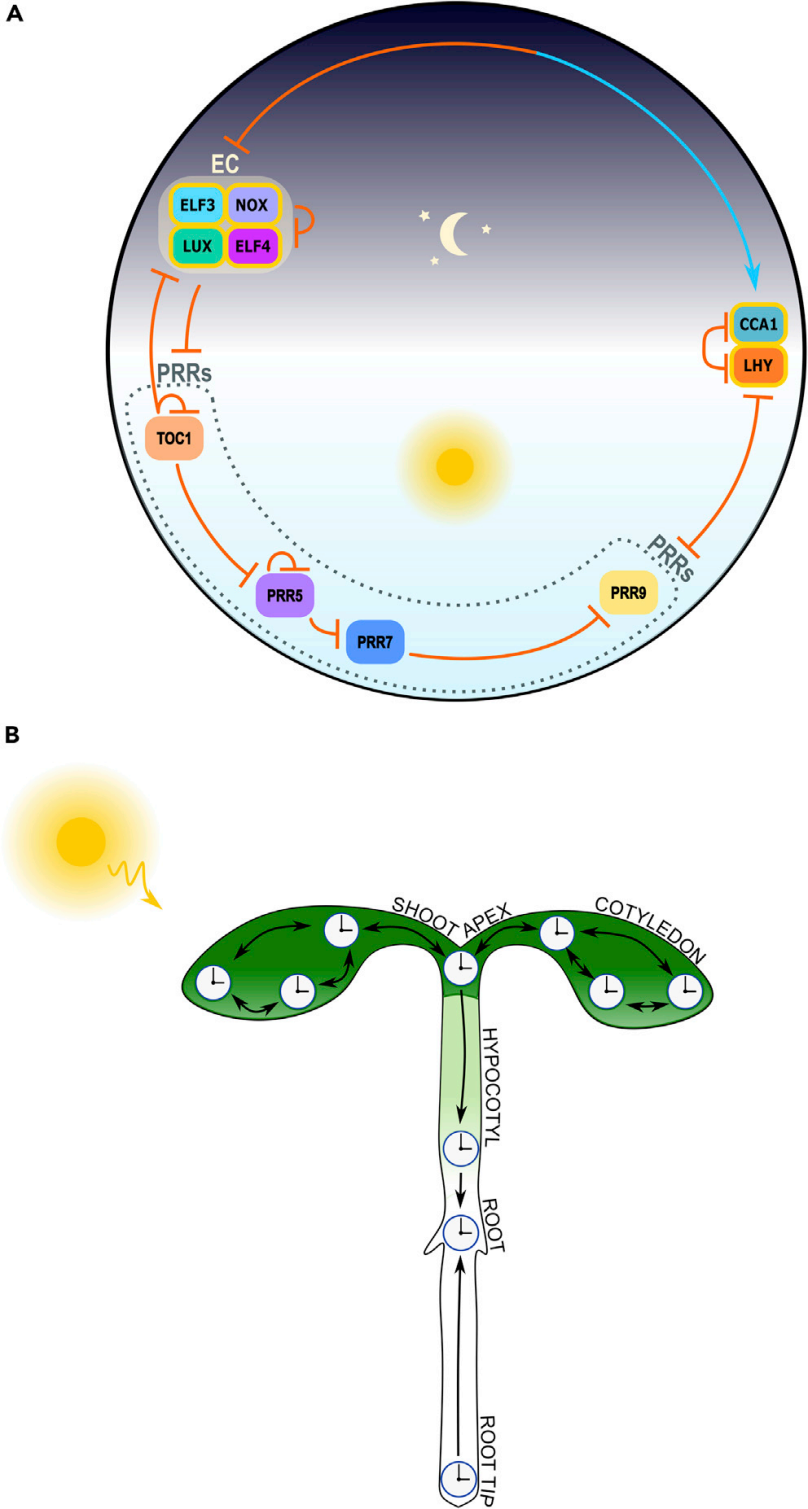
The circadian clock in plants (A) Simplified molecular clock mechanism.At dawn, CCA1 and LHY are expressed and, in addition to repressing themselves via G-box and 5A motifs (Schaffer et al., 1998; Wang and Tobin, 1998; Adams et al., 2015), heterodimerize, allowing binding to ‘Evening Element’ (EE) motifs (Harmer et al., 2000; Lu et al., 2009; Yakir et al., 2009; Nagel et al., 2015; Kamioka et al., 2016) in promoter regions of *TOC1* (*PRR1*), other *PRR* family genes and *GI* (not shown), repressing them to limit their expression to later in the day. In turn, the PRR proteins repress *CCA1* and *LHY* in a time-delayed fashion, via expression of the sequentially repressive *TOC1*, *PRR5*, *PRR7* and *PRR9* throughout the day (Nakamichi et al., 2010) which feedback upon and temporally limit *CCA1* and *LHY* expression to the morning. Expression of *LUX*, *ELF3* and *ELF4* is restricted, due to repression from CCA1:LHY and TOC1, to the evening, when they form the ‘Evening Complex’ (EC) (Portolés and Más, 2010; Li et al., 2011; Nusinow et al., 2011; Huang et al., 2012). The EC represses *PRR7*, *PRR9*, itself and *GI* (not shown) progressing throughout the night resulting in indirect and direct, via the LUX homologue, NOX (Dai et al., 2011) promotion of *CCA1* expression by subjective dawn (Chow et al., 2012; Mizuno et al., 2014). This core clock is supplemented with activatory input (not shown) from LWD1, RVE8 and LNK proteins, as well as indirectly from *GI* (Martin-Tryon et al., 2007; Farinas and Mas, 2011; Rawat et al., 2011; Wang et al., 2011; Lu et al., 2012; Hsu et al., 2013; Rugnone et al., 2013; Xie et al., 2014; Wu et al., 2016). Components are positioned on a 24-hr dial (top is midnight) corresponding to approximate time of peak expression. Gold borders indicate protein complexes. Positive and negative regulation signified by blue pointed arrows and orange flat-headed arrows, respectively. Where regulation is bidirectional, double-ended arrows are used. (B) Circadian organization. The main plant clocks studied: cotyledons, shoot apex, hypocotyl, root, and root tip are shown, along with their interactions. Intrinsic photoentrainability of all plant clocks to sunlight zeitgebers, along with further coordination received from shoot apex and root tip clocks and localized coupling, allow plant clocks to operate in a decentralized hierarchy. Arrows represent entraining influence within the circadian hierarchy and coupling between cellular clocks.

The core plant clock TTFLs (Figure 4A) primarily feature *CCA1*, *LHY*, *TOC1*, *GI*, *PRRs* and an evening-expressed clock gene ensemble, the ‘Evening Complex’ (EC), with additional activatory input from *LWD1*, *RVE8*, and *LNK*. These constitute a complex network, interacting to generate ∼24-hr rhythms in their own and downstream CCG expression, providing circadian timing for fundamental processes, from growth to photosynthesis (Dowson-Day and Millar, 1999; Rascher et al., 2001).

## Plant clocks operate in a locally coupled and decentralized hierarchy

In contrast to mammalian clocks, which are coordinated centrally by the SCN, evidence suggests plant clocks operate as a decentralized hierarchy of locally coupled oscillators.

**Figure 5:**
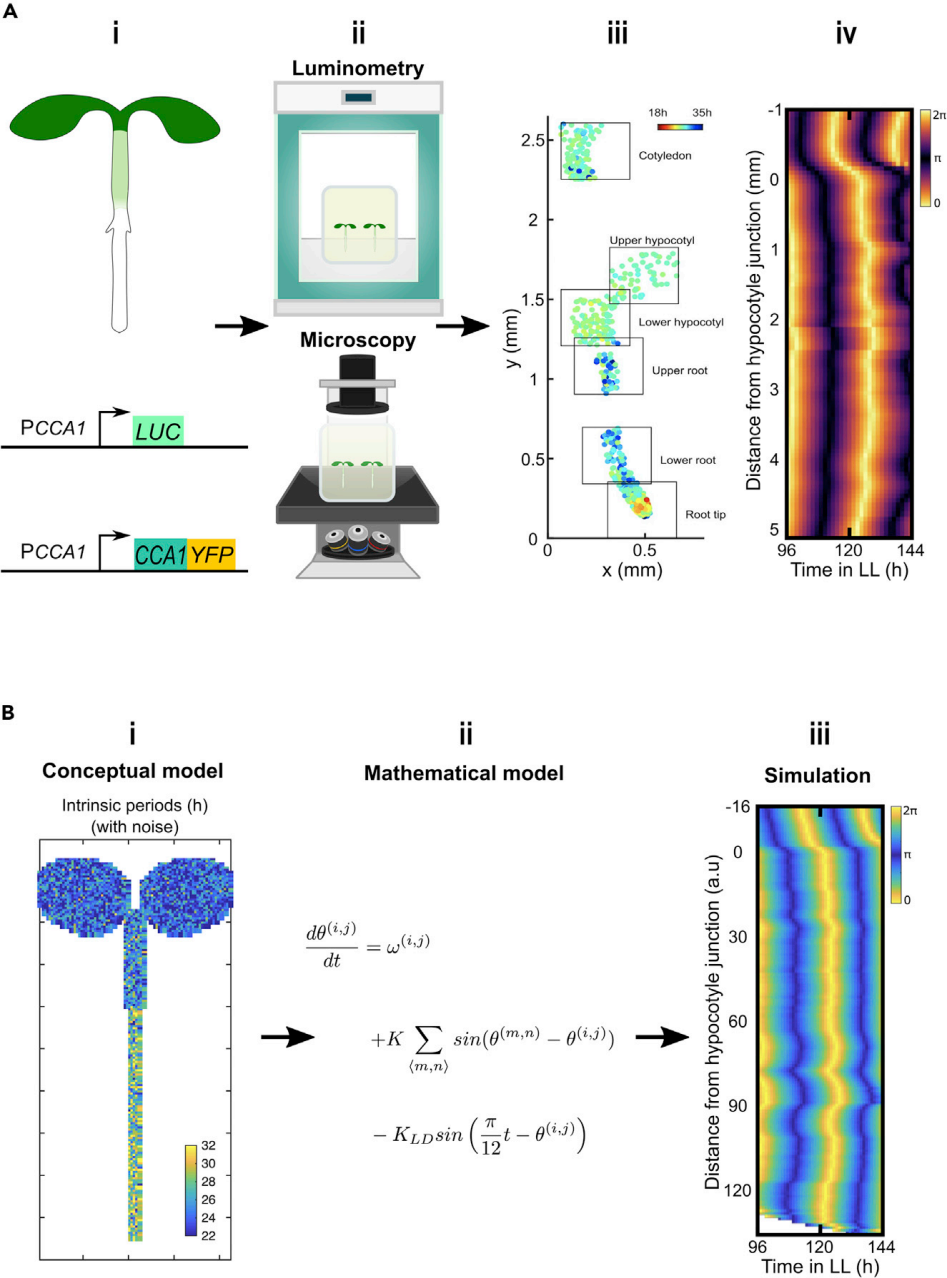
Studying coupling in plants (A) Key experimental techniques. (i) *Arabidopsis thaliana* is the predominant model for studying coupling in plants, via a variety of bioluminescent and fluorescent transcriptional and translational clock gene reporters (examples shown). (ii) These can be observed using time-lapse organism-level luminometry (bioluminescent reporters) or single-cell-level microscopy (fluorescent reporters) under constant zeitgeber conditions. Light is supplied as necessary for entrainment and photosynthesis. (iii) Example single-cell confocal data showing CCA1-YFP reporter fluorescence in an *Arabidopsis* seedling. Dots represent single cells, colour-coded by period length, in the spatial context of a seedling (figure adapted, under Creative Commons Attribution Licence version 4.0 (CC BY 4.0), from Gould, P. D. et al. (2018) ‘Coordination of robust single cell rhythms in the *Arabidopsis* circadian clock via spatial waves of gene expression’, *eLife*, 7. https://doi.org/10.7554/eLife.31700. Copyright 2018 by Gould et al.). (iv) Example kymograph of *GI::LUC* bioluminescent reporter expression across a longitudinal section of *Arabidopsis* seedling hypocotyl and root, under constant light (LL), colour-coded by circadian phase. These data demonstrate how single-cell- and organism-level circadian dynamics can be tracked with high spatial resolution in living plants, revealing different intrinsic periods and spatial waves in the absence of rhythmic light (figure adapted, under Creative Commons Attribution Licence version 4.0 (CC BY 4.0), from Greenwood, M. et al. (2019) ‘Coordinated circadian timing through the integration of local inputs in *Arabidopsis thaliana*’, *PLoSBiology*, 17(8), p. e3000407. https://doi.org/10.1371/journal.pbio.3000407. Copyright 2019 by Greenwood et al.). (B) Key theoretical techniques (approach from Greenwood et al. (2019)). (i) Conceptual framework within which plant clock simulations can be made, using data-driven color coding of each pixel by intrinsic period (figure adapted, under Creative Commons Attribution Licence version 4.0 (CC BY 4.0), from Greenwood, M. et al. (2019) ‘Coordinated circadian timing through the integration of local inputs in *Arabidopsis thaliana*’, *PLoSBiology*, 17(8), p. e3000407. https://doi.org/10.1371/journal.pbio.3000407. Copyright 2019 by Greenwood et al.). (ii) Pixels are described, via differential equations, as locally coupled Kuramoto phase oscillators. (iii) Simulated kymograph of hypocotyl and root clock gene dynamics under constant light (LL) recapitulates experimental data (iv), indicating plant clocks behave as locally coupled phase oscillators (figure adapted, under Creative Commons Attribution Licence version 4.0 (CC BY 4.0), from Greenwood, M. et al. (2019) ‘Coordinated circadian timing through the integration of local inputs in *Arabidopsis thaliana*’, *PLoSBiology*, 17(8), p. e3000407. https://doi.org/10.1371/journal.pbio.3000407. Copyright 2019 by Greenwood et al.).

**Figure 6:**
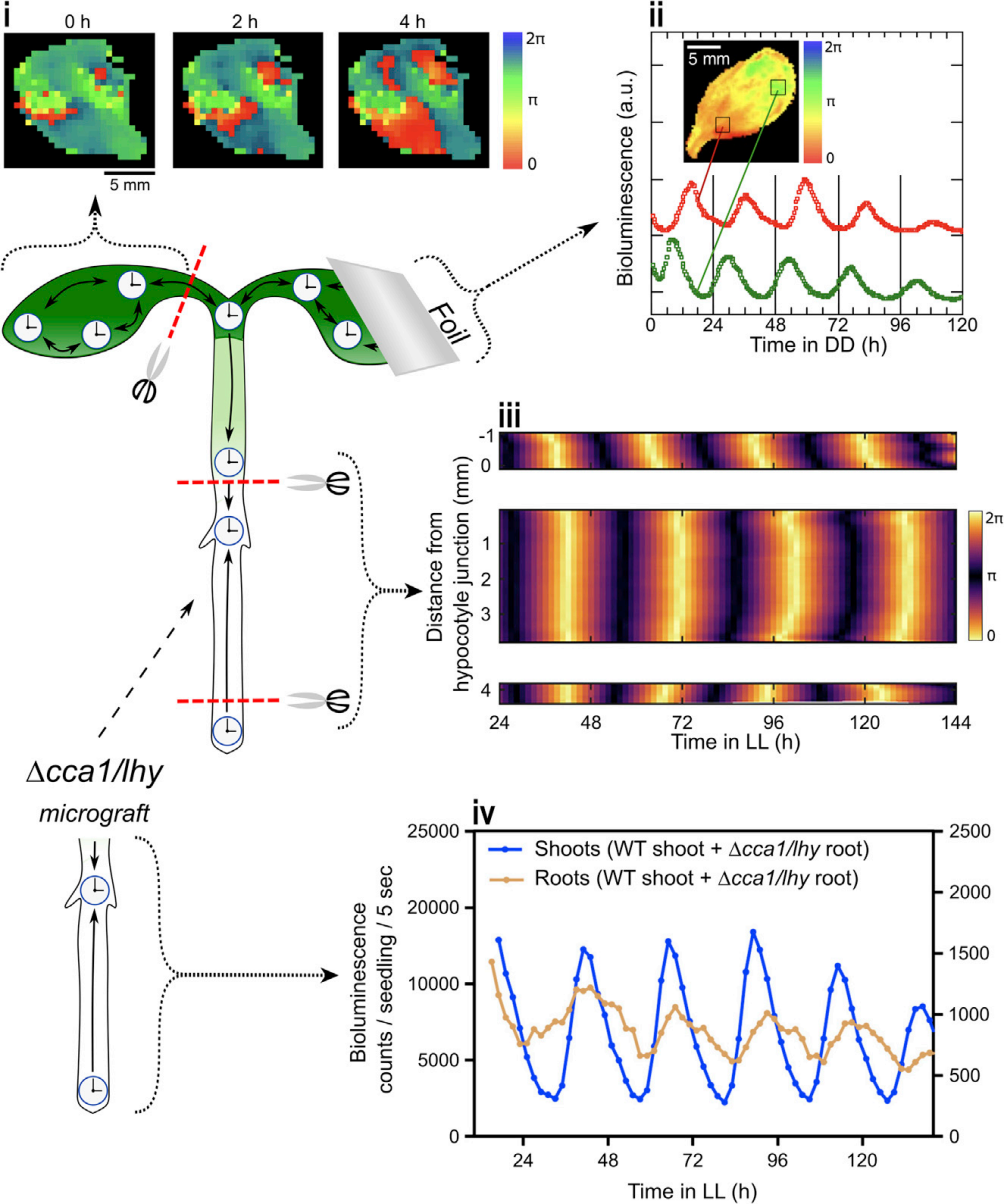
Evidence for coupling in plants (i**)** Bioluminescence imaging data showing spiral patterns of *CCA1::LUC* expression in a detached *Arabidopsis* leaf with local coupling (figure adapted, with permission, from Fukuda, H. et al. (2007) ‘Synchronization of plant circadian oscillators with a phase delay effect of the vein network’, *Physical review letters*, 99(9), p. 098102. https://doi.org/10.1103/PhysRevLett.99.098102. Copyright 2007 by the American Physical Society). (ii) *CCA1::LUC* bioluminescence traces of antiphase-entrained (by alternately covering with foil) *Arabidopsis* cotyledon halves (red and green lines, respectively), gradually shifting to an intermediate phase, due to local circadian coupling under constant dark (DD). Colour-coded by circadian phase (figure adapted, with permission, from Fukuda, H. et al. (2007) ‘Synchronization of plant circadian oscillators with a phase delay effect of the vein network’, *Physical review letters*, 99(9), p. 098102. https://doi.org/10.1103/PhysRevLett.99.098102. Copyright 2007 by the American Physical Society). (iii) *GI::LUC* bioluminescence kymograph data of *Arabidopsis* seedling sections shows spatial waves of clock gene expression persist under constant light (LL) when long-distance signaling is severed, indicative of localized coupling. Colour-coded by circadian phase (figure adapted, under Creative Commons Attribution Licence version 4.0 (CC BY 4.0), from Greenwood, M. et al. (2019) ‘Coordinated circadian timing through the integration of local inputs in *Arabidopsis thaliana*’, PLoS Biology, 17(8), p. e3000407. https://doi.org/10.1371/journal.pbio.3000407. Copyright 2019 by Greenwood et al.). (iv) *TOC1::LUC* bioluminescence traces showing rescue of *Δcca1/lhy* arrhythmic mutant *Arabidopsis* rootstocks (brown lines) grafted onto wild type shoot scions (blue lines) under constant light (LL), demonstrating coupling is hierarchical between these plant organs (figure adapted, with permission, from Takahashi, N. et al. (2015) ‘A hierarchical multi-oscillator network orchestrates the *Arabidopsis* circadian system’, *Cell*, 163(1), pp. 148–159. https://doi.org/10.1016/j.cell.2015.08.062. Copyright 2015 by Elsevier).

Plant clock studies have greatly benefited from development of real-time fluorescent and bioluminescent reporters (Figure 5A) and were notable for early use of such tools in probing in vivo temporal gene expression dynamics (Millar et al., 1992; Siebke and Weis, 1995; Michelet and Chua, 1996; Bognár et al., 1999). Due to technical challenges associated with prolonged live plant cell imaging, single-cell level studies are less abundant than in mammals. However, the intrinsic photoentrainability of plant tissues is exploitable for studying plant clock coupling in unique, ingenious ways (Figure 6). For example, by alternately covering parts of reporter-carrying plants, spatially distinct regions of the same plant are entrainable in antiphase (Figure 6ii). If circadian coupling is present, phase shifts are expected, as neighboring regions resynchronize on return to constant conditions. Thain et al. pioneered this technique in 2000 with transcriptional bioluminescent reporters of the CCGs *PHB*, *CHS* and *CAB* in *Nicotiana tabacum* and *Arabidopsis thaliana* (*Arabidopsis*) (Thain et al., 2000). However, they did not detect phase shifts in neighboring regions up to 70 hr after returning to constant conditions, suggesting an absence of coupling (Thain et al., 2000). This was likely limited by the time and spatial scales (i.e., opposite cotyledons) examined. Indeed, a later study applying the same technique to leaf sub-regions of mature *Arabidopsis CCA1::LUCIFERASE* transcriptional reporter plants (Doyle et al., 2002; Nakamichi et al., 2004), showed phase shifts occurring within 120 hr (Figure 6ii) (Fukuda et al., 2007). Such shifts, predicted to result in complete resynchronization after 100 circadian cycles, indicate weak coupling (Fukuda et al., 2007).

Coupling is further evidenced by spatiotemporal waves and spirals of clock gene expression (Figure 6I), spanning intact and detached leaves as well as roots. These have been described in not only *Arabidopsis*, but also *Kalanchoe daigremontiana* (mother of thousands) and the agriculturally relevant *Lactuca sativa L.* (lettuce) (Rascher et al., 2001; Fukuda et al., 2007; Fukuda et al., 2012; Wenden et al., 2012; Ukai et al., 2013). In detached leaves, waves propagate from fewer points (Fukuda et al., 2007), while clock phase appears to be delayed by the veins, implicating vascular network involvement (Fukuda et al., 2007; Ukai et al., 2013). Mathematical modeling has helped explain these phenomena. A relaxation oscillator model has proposed spatiotemporal waves stem from intercellular dephasing and variability, resulting from stochastic noise within a system of weakly coupled circadian oscillators (Beck et al., 2001). Alternative methods, based on coupled Stuart-Landau equations numerically simulated with the fourth-order Runge-Kutta method, have also found these dynamics are consistent with weak clock coupling (Fukuda et al., 2007).

Some evidence, however, such as the desynchronization of guard cells relative to non-stomatal cells under constant conditions (Yakir et al., 2011), suggests intercellular coupling is not ubiquitous among plant cells. While this may be a peculiarity of guard cells, perhaps due to loss of plasmodesmata during development (Palevitz and Hepler, 1985), such desynchronization is not necessarily incompatible with the existence of weak coupling. Indeed, sufficiently weak coupling can have negligible effects over experimental timescales, during which stochastic modeling predicts noisy plant oscillators will partially desynchronize (Guerriero et al., 2012)

The plant clock coupling so far discussed appears similar to that found among mammalian peripheral clocks (Nagoshi et al., 2004; Welsh et al., 2004; Yoo et al., 2004; Rougemont and Naef, 2007), thus, one might wonder whether these similarly exist in a hierarchy. Though roots can produce circadian rhythms while detached from shoots (Bordage et al., 2016; Li et al., 2020c), disrupting capacity for rhythmic shoot signaling with chemical perturbation or decapitation in *Arabidopsis* seedlings, has nevertheless been shown to impact root clock dynamics, consistent with a shoot-dominant hierarchy (James et al., 2008; Bordage et al., 2016). Indeed, the shoot and shoot apex are noteworthy for their potential to coordinate root clocks in *Arabidopsis* seedlings, possibly via ELF4 trafficking (Takahashi et al., 2015; Chen et al., 2020). Blocking shoot apex signals via laser microdissection, chemical perturbation,and *elf4* or plasmodesmata mutation, result in root clock damping (Takahashi et al., 2015; Chen et al., 2020). Micrografting wild type and *ELF4-*overexpressing shoot scions onto arrhythmic rootstocks rescues root rhythms (Figure 6iv) (Takahashi et al., 2015; Chen et al., 2020), while opposite procedures fail to do so (Takahashi et al., 2015). Although a recent study shows that rhythmic light, exposed to roots, can override putative shoot signals, it is not yet clear which might take precedence under true physiological conditions; indeed, it is probable both play an important role in coordinating root clocks (Bordage et al., 2016). Thus, current evidence supports hierarchical coupling between plant clocks, with potential for an SCN-like role of the shoot and shoot apex.

Ingenious use of split-luciferase reporters (Paulmurugan et al., 2002) allows tissue-specific clock reporting in *Arabidopsis* seedlings. This has revealed distinct *TOC1* dynamics in vasculature clocks, uncovering an additional, tissue-level circadian hierarchy (Endo et al., 2014). Compared to isolated mesophyll, isolated vascular tissues maintain robust circadian rhythms under constant light, indicating stronger coupling (Endo et al., 2014). Furthermore, tissue-specific perturbations of vasculature and mesophyll clocks reveal asymmetric influence of vasculature over mesophyll, again consistent with a circadian hierarchy (Endo et al., 2014).

Further similarities between plant and mammalian circadian systems are apparent from single-cell fluorescent reporter studies. For instance, shoot apex clocks show highly density-dependent synchrony of rhythms (Takahashi et al., 2015), akin to coupled mammalian clocks (Webb et al., 2009; Noguchi et al., 2013). Fitting these data to predictive mathematical models using barycentric coordinates for a higher dimensional space assigns high weightings to adjacent cells, comparable to those obtained from fitting Kuramoto and Rössler coupled oscillator models (Kuramoto, 1975; Rössler, 1976), confirming significant coupling between shoot apex cells. Repeating such methods on vascular and mesophyll cells suggests they have intermediate and weak coupling, respectively, consistent with prior work (Fukuda et al., 2007; Endo et al., 2014). A study of single mesophyll cells has also revealed that although noisy mesophyll clocks phase disperse under constant light, they nevertheless exhibit spatially correlated phases (Muranaka and Oyama, 2016). This is not unlike hepatocyte clocks (Guenthner et al., 2014) and is consistent with weak coupling (Muranaka and Oyama, 2016).

The above suggests that plant clocks, like those of mammals, exist in a circadian hierarchy. In mammals, evidence supports a *centralized* hierarchy, that is, the strongly coupled SCN coordinates weakly coupled peripheral tissues (Pando et al., 2002; Welsh et al., 2004; Rougemont and Naef, 2007; Koronowski et al., 2019). However, hierarchical coupling seems more widespread in plants, leading to the question: does this indicate a *decentralized* hierarchy, with multiple coordination centers, in contrast to that of mammals? Recent advances in single-cell imaging have helped tackle this question (Gould et al., 2018). In 2018, utilizing novel confocal imaging methods, Gould et al. tracked the circadian dynamics of an order of magnitude more single cells than had been possible before, for long periods across whole plants. Although the study did not explicitly investigate the reportedly SCN-like shoot apex (Takahashi et al., 2015), previous observations of robust oscillations in excised hypocotyls and cotyledons (Takahashi et al., 2015) could be recapitulated alongside waves of clock gene expression in the root (Fukuda et al., 2012). Simulations with a coupled Kuramoto phase oscillator model attributed this to longer root clock periods relative to the shoot and interestingly, root tip, resulting in waves of phase-resetting from these points. Although the shoot has previously been identified as a strongly coupled potential circadian pacemaker in plants (James et al., 2008; Takahashi et al., 2015), it appears strong coupling, typified by an increasing order parameter over time (Kuramoto, 1984), also exists in the root tip where cell density is correspondingly high.

Most recently, in continuation of this work, Greenwood et al. have investigated the levels of plant clock coordination further (Greenwood et al., 2019). Applying the same techniques, utilizing a *GI::LUCIFERASE* reporter, has revealed disparate circadian phases in *Arabidopsis* cotyledons, hypocotyls, roots, and root tips under entraining and constant conditions. This suggests different endogenous clock periods in different plant organs, aligned with earlier observations (Thain et al., 2002). As reported previously, these phase differences induce spatiotemporal waves up and down the root (Gould et al., 2018) as well as from the cotyledon tips and roots toward the hypocotyl which persist even when connections between organs are severed (Figure 6iii). This implies long-distance signaling is not necessary for coordinating such phenomena. Instead, the previous coupled phase oscillator model (Gould et al., 2018) shows the entrainment behaviors can be explained and spatiotemporal waves replicated through the existence of local cell-to-cell coupling and endogenous period differences (Figure 5B). Thus, observing plants in such detail has revealed a hierarchical, yet *decentralized* circadian oscillator network (Figure 4B) (Endo, 2016). This features local coordination with strongly influential coupling in the shoot apex and root tip, intermediate in the shoots and developing vasculature and weakest in the mesophyll and roots. However, despite this abundant evidence for coupling in plants, the exact molecules that might mediate this coupling are, relative to mammals, largely unknown.

## The circadian clock in cyanobacteria

**Figure 7:**
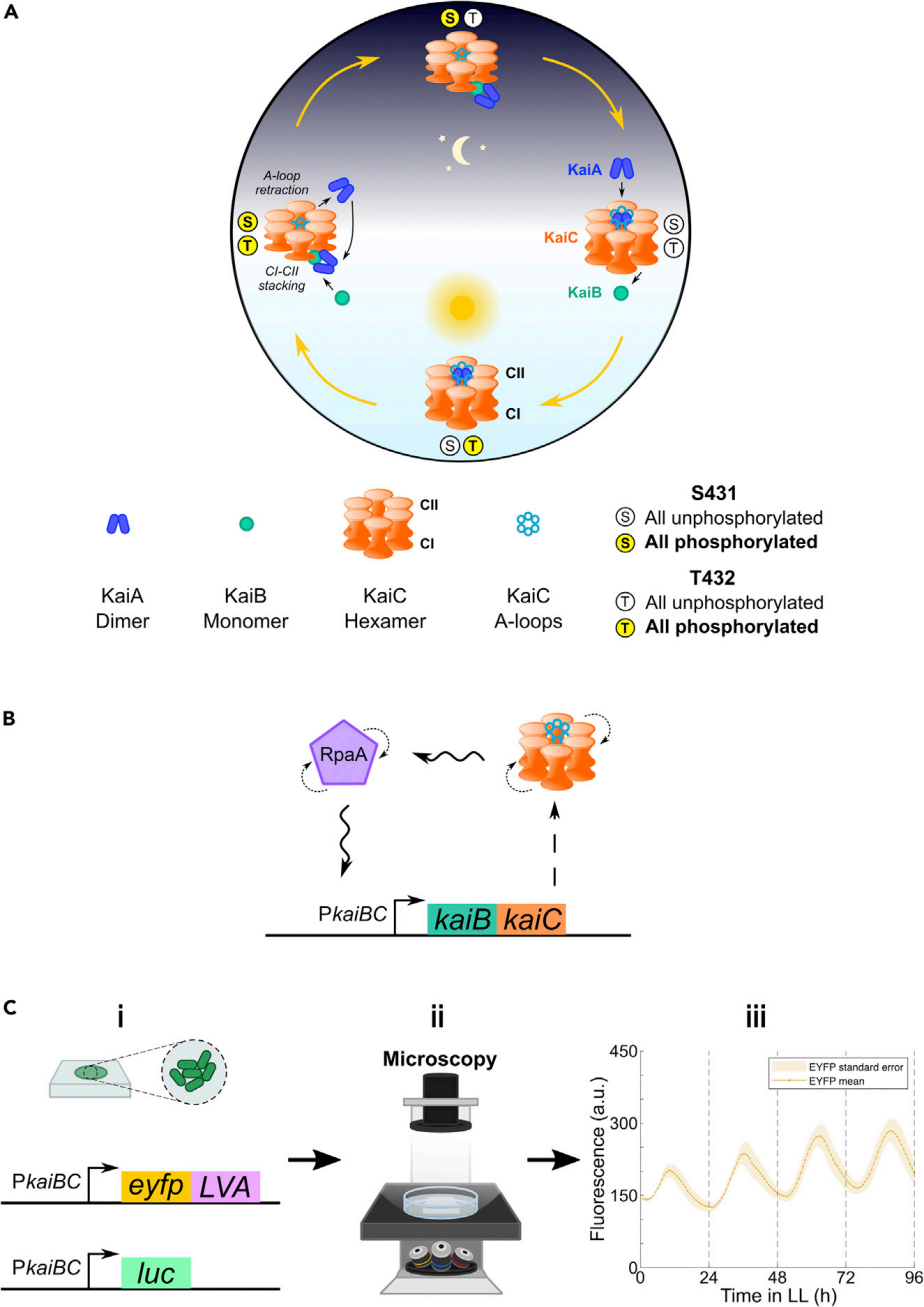
The circadian clock in cyanobacteria (A) Simplified molecular mechanism. KaiC, a homohexameric protein, forms what has been dubbed a “double doughnut” structure (Cohen and Golden, 2015), comprising two rings, CI and CII (Pattanayek et al., 2004). KaiA is a homodimeric protein (Uzumaki et al., 2004), while KaiB transitions (not shown) between homotetrameric, homodimeric, and monomeric “fold-switched” states (Hitomi et al., 2005). A-loops extend from the C-terminal end of CII (Pattanayek et al., 2004), which are bound to and stabilized by KaiA in the non-phosphorylated KaiC state (right), inducing KaiC autophosphorylation during subjective day (Kim et al., 2008). This occurs sequentially at two residues located on the CII half of each KaiC subunit: first at T432 (bottom) and then S431 (left) (Rust et al., 2007), the latter resulting in ring-ring stacking, exposing a KaiB-binding site on the CI ring (Chang et al., 2012). KaiB, having undergone a transition to its monomeric “fold-switched” state, binds CI, along with KaiA (Chang et al., 2015). This sequestration of KaiA away from the A-loops creates a negative feedback loop (Clodong et al., 2007), triggering A-loop retraction and nighttime KaiC autophosphatase activity: starting with the T432 residues (top) followed by the S431 residues, eventually returning to a non-phosphorylated state (right), ready to bind KaiA once more (Kim et al., 2008; Chang et al., 2012). Processes positioned on a 24-hr dial (top is midnight) corresponding to approximate timing of occurrence. (B) Post-translational oscillator (PTO) to transcriptional-translational feedback loop (TTFL) transduction. KaiC phosphorylation state signals are transduced, affecting phosphorylation states of other transcription factors and intermediaries including the master transcriptional regulator of the *S. elongatus* circadian clock, RpaA (Markson et al., 2013). Oscillations in KaiC phosphorylation indirectly induce 24-hr oscillations in RpaA phosphorylation and regulatory activity. In-turn, RpaA supplements the core circadian PTO with a TTFL, regulating the *kaiB* and *kaiC* shared promoter, P*kaiBC* in a circadian manner. Dashed line indicates expression, small dotted lines indicate phosphorylation cycles, and wavy lines indicate transduction of oscillatory phosphorylation signal. (C) Studying the clock in cyanobacteria. (i) *S. elongatus* PCC 7942 is the predominant model for studying clock coupling in cyanobacteria, via transcriptional bioluminescent and fluorescent reporters, driven by the robustly circadian promoter P*kaiBC*. Fluorescent reporters feature an SsRA-LVA (Andersen et al., 1998) degradation tag to prevent buildup. (ii) Cells are usually observed on agarose pads, using time-lapse fluorescence or bioluminescence microscopy. Light is supplied as necessary for entrainment and photosynthesis. (iii) Example time-lapse P*kaiBC*::EYFP-LVA fluorescence trace of *S. elongatus* strain JRC35 (Chabot et al., 2007) under constant light (LL), demonstrating circadian dynamics from single cells within colonies (Chris N. Micklem, unpublished data). Yellow line is the mean and yellow area represents the standard error.

The circadian clocks of cyanobacteria, a diverse phylum of photosynthetic bacteria, are possibly the simplest yet discovered. The domesticated *Synechococcus elongatus* isolate, PCC 7942 has become the established model for cyanobacterial chronobiology (Kondo et al., 1993). A key difference between cyanobacterial and eukaryotic clocks is that the former, at their core, constitute post-translational oscillators (PTOs), while the latter comprise TTFLs. In *S. elongatus* this core PTO is made up of three proteins: KaiA, KaiB, and KaiC (Ishiura et al., 1998) which, even *in vitro*, maintain ∼24-hr rhythms in KaiC phosphorylation (Figure 7A) (Nakajima et al., 2005; Rust et al., 2007). KaiC phosphorylation state signals are transduced, influencing other transcription factors and intermediaries, including the master transcriptional regulator of the *S. elongatus* clock, RpaA (Figure 7B) (Markson et al., 2013). RpaA supplements the core PTO with a TTFL, regulating the shared promoter of *kaiB* and *kaiC*, P*kaiBC* in a circadian manner. In this regard, while cyanobacterial clocks possess a self-sustaining PTO at their core, TTFLs are vital for enacting their physiological outputs. Under RpaA regulation, P*kaiBC* shows robust ∼24-hr oscillations in activity, indirectly representing the state of KaiC and the circadian clock in *S. elongatus*.

Though *S. elongatus* is taken as representative of cyanobacterial timekeepers, it is important to recognize fundamental differences do exist in other cyanobacteria. The significantly smaller ocean-dwelling cyanobacterium *Prochlorococcus marinus* operates with a simpler, KaiA-free circadian timekeeper (Holtzendorff et al., 2008). The absence of KaiA removes negative feedback from the system, preventing sustained KaiC phosphorylation cycles under constant light (Clodong et al., 2007; Chew et al., 2018). This results in a so-called ‘hourglass’ timer incapable of sustaining oscillations in the absence of zeitgebers. Recent research suggests a conserved set of genes including *cpmA*, *ircA*, *lpdA*, *rpaB*, and *sasA*, in addition to those already discussed, may contribute to this core ‘hourglass’ timing mechanism (Schmelling et al., 2017; Kawamoto et al., 2020). As we will discuss later, such timekeepers might be particularly beneficial for smaller, noise-prone cells with lower protein copy numbers and may be pervasive throughout prokarya (Schmelling et al., 2017).

It should be noted that while cyanobacteria have been considered to possess the archetypal bacterial clock, this notion has recently been challenged by the discovery of an oscillator in *Bacillus subtilis* carrying all the hallmarks of a circadian clock (Eelderink-Chen et al., 2021). The molecular mechanism for this clock remains to be elucidated, however, the lack of *kai* homologues in *B. subtilis* as well as the presence of Per-Arnt-Sim domains in its circadian genes, akin to eukaryotes, carries interesting implications for circadian clock origins. Furthermore, this work is significant in its demonstration that circadian timekeeping among unicellular organisms is not limited to photoautotrophs.

## Clocks of unicellular cyanobacteria show exceptional robustness in the absence of coupling

Cyanobacterial clock reporters are predominantly driven by P*kaiBC* (Figure 7C). Though originally only bioluminescent reporters existed (Kondo et al., 1993), fluorescent reporters have become increasingly popular, particularly for single-cell-level observation (Chabot et al., 2007; Martins et al., 2016; Chew et al., 2018; Arbel-Goren et al., 2021).

**Figure 8:**
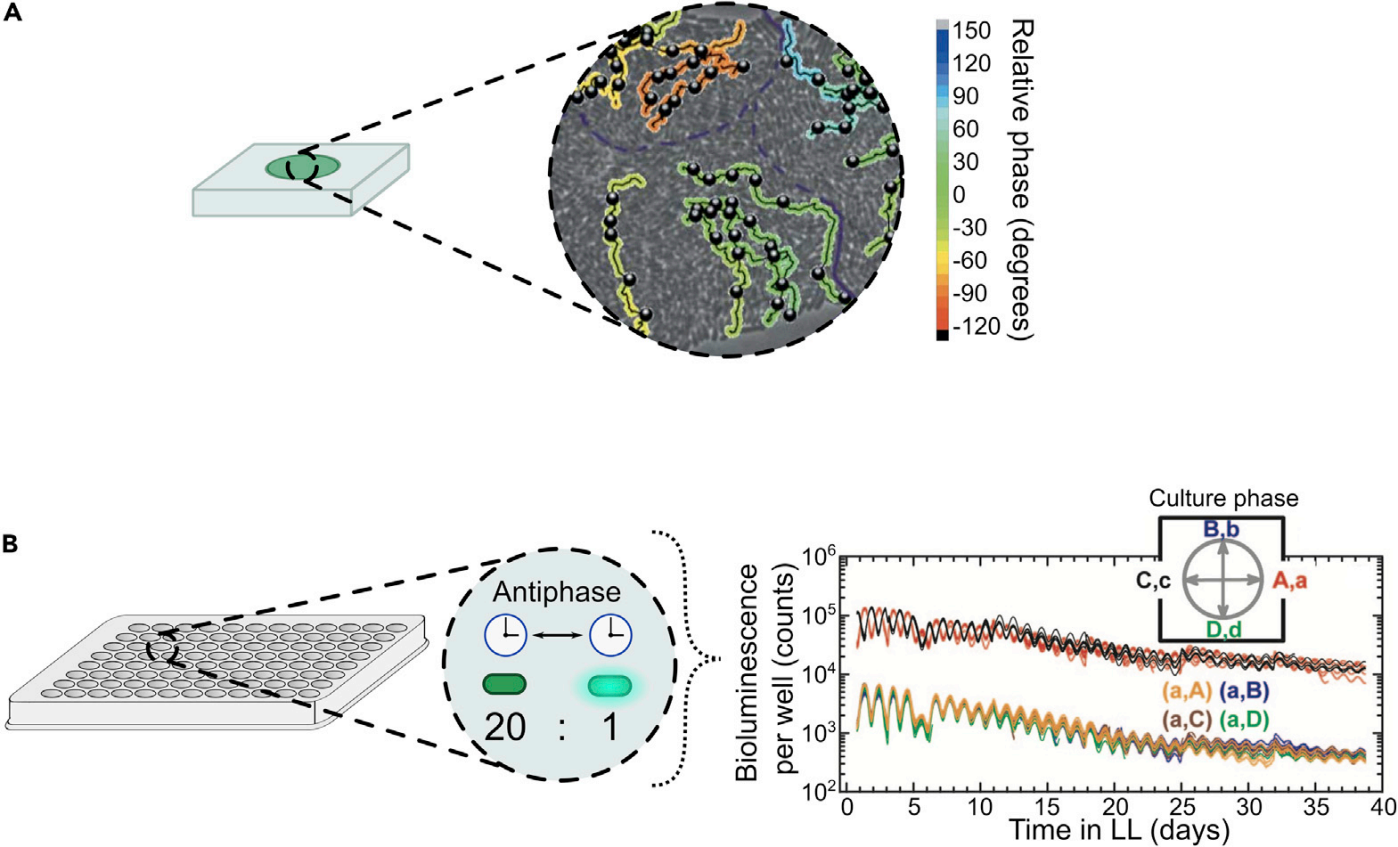
Evidence for no coupling in unicellular cyanobacteria (A**)** P*kaiBC*::LUC *S. elongatus* bioluminescence image shows clock phases of agarose pad-grown *S. elongatus* cells are conserved within lineages but not between lineages in close proximity. This suggests there is no coupling of sufficient strength to synchronize adjacent lineages. Lineages track the path of individual cells over time and are colour-coded by circadian phase. Black circles indicate cell divisions. Purple lines indicate microcolony boundaries (figure adapted, with permission, from Mihalcescu, I., Hsing, W. and Leibler, S. (2004) ‘Resilient circadian oscillator revealed in individual cyanobacteria’, *Nature*, 430(6995), pp. 81–85. https://doi.org/10.1038/nature02533. Copyright 2004 by Macmillan Magazines Ltd). (B) P*kaiBC*::LUC *S. elongatus* bioluminescence data shows reporter cells co-cultured with a 20-fold larger population of differentially entrained non-reporter cells do not shift in phase toward that of the dominant population, even after several weeks under constant light (LL), indicating no significant coupling between the subpopulations. Cells are entrained in four phases (A, B, C and D) 6 hr apart. Upper-case letters indicate the non-luminescent, 20-fold majority population phase in a culture. Lower-case letters indicate the luminescent reporter-carrying minority population phase in a culture. Single-phase control cultures A,a and C,c have opposite phases (red and black lines, respectively) showing successful antiphase entrainment. Luminescent reporter-carrying minority strains in phase A exhibit negligible phase shifts after weeks of co-culture with non-luminescent majority strains in phases A (control), B, C and D (cultures: a,A; a,B; a,C; a,D and colors: yellow, blue, brown, green, respectively), indicating insignificant coupling (figure adapted, under the exclusive PNAS License to Publish, from Amdaoud, M. et al. (2007) ‘Cyanobacterial clock, a stable phase oscillator with negligible intercellular coupling’, *Proceedings of the National Academy of Sciences of the United States of America*, 104(17), pp. 7051–7056. https://doi.org/10.1073/pnas.0609315104. Copyright 2007 by National Academy of Sciences).

Current understanding of clock coupling in cyanobacteria comes from three main studies (Mihalcescu et al., 2004; Amdaoud et al., 2007; Arbel-Goren et al., 2021). Mihalcescu, Hsing, and Leibler first reported in 2004 that no coupling exists between *S. elongatus* PCC 7942 clocks, concluded through tracking oscillations in P*kaiBC*-driven bioluminescence of agarose pad-grown single cells (Mihalcescu et al., 2004). A degree of heterogeneity in circadian phase within microcolonies was observed (Figure 8A). Closer inspection revealed this heterogeneity existed even between spatially close cell lineages, while cells within lineages remained largely synchronous. This implies *S. elongatus* PCC 7942 clock phase is maintained through lineage, by exceptionally robust, uncoupled clocks.

Later work from Amdaoud et al. in 2007 further suggested that *S. elongatus* PCC 7942 lacked coupling (Amdaoud et al., 2007). This study, also tracking P*kaiBC*-driven reporter bioluminescence, searched for phase shifts when co-cultured with an antiphase-entrained reporterless strain in 96-well-plate liquid cultures (Figure 8B). A noisy phase oscillator model informing experimental design, suggested phase shifts in reporter populations comprising 1/20th a co-culture should be detected in the presence of significant coupling. Cultures were entrained in four phases (A, B, C, and D) 6 hr apart and, from these, co-cultures were set up containing 1/20th a bioluminescent reporter strain and the remainder a non-luminescent strain in each of the four phases (Figure 8B: cultures a,A; a,B; a,C and a,D). Though single-phase control co-cultures (Figure 8B: cultures A,a and C,c) confirmed differential entrainment had been successful, mixed-phase co-cultures (Figure 8B: cultures a,A; a,B; a,C and a,D) all remained in the same phase, indicating an inability for the majority phase to affect the minority through coupling. A coupling strength could nonetheless be estimated, suggesting a minimum of ∼500 days for co-culture resynchronization. This was again consistent with insignificant coupling between *S. elongatus* PCC 7942 clocks, contrary to the eukaryotes discussed. However, as individual KaiC assemblages can be viewed as individual clocks, these are essentially coupled *intracellularly*: via a shared KaiA pool (Clodong et al., 2007; Chew et al., 2018) and KaiC monomer shuffling (Clodong et al., 2007; Ito et al., 2007), which average over the KaiC hexamers, resulting in relatively synchronized phosphorylation cycles.

Excitingly, in contrast to the unicellular cyanobacteria so far discussed, a study has most recently found evidence for coupling in the filamentous multicellular cyanobacterium *Anabaena* sp. PCC 7120 (Arbel-Goren et al., 2021). Arbel-Goren et al. found that individual cells expressing a fluorescent *pecB* CCG reporter within *Anabaena* sp. PCC 7210 filaments were significantly more synchronous than those between filaments, as determined by their order parameter, *R* (Garcia-Ojalvo et al., 2004). The calculated spatial autocorrelation of clock phase could not be explained by heritability alone, indicating cell-to-cell coupling may be involved. This was strongly supported by genetic perturbation of the septal junctions connecting adjacent *Anabaena* sp. PCC 7210 cells, which resulted in much faster decay in spatial autocorrelation and significantly reduced order parameter (Arbel-Goren et al., 2021). Thus, these latest results have demonstrated that circadian coupling in cyanobacteria can exist, at least via direct cytoplasmic connections in filamentous species.

Given the recentness of the identification of a circadian clock in the non-photosynthetic bacterium *B. subtilis* (Eelderink-Chen et al., 2021), its robustness and potential coupling have yet to be explored. However, Eelderink-Chen et al. noted when reporting their discovery that circadian rhythms were uniquely found in pellicle biofilm-forming cultures (Eelderink-Chen et al., 2021). Considering the multicellular nature of biofilms and the role of intercellular coordination mechanisms in their formation (Duanis-Assaf et al., 2015; Omer Bendori et al., 2015), it is possible *B. subtilis* clocks are also coupled. Indeed, this will be a fascinating angle for future research.

## Advantages of varied circadian coupling

We have so far visited a range of circadian clocks, exploring both theoretical and experimental evidence for coupling within different circadian systems. We have learnt that in mammals, coupling and as a result, heterogeneity among clocks varies between different organs, which form an SCN-directed centralized hierarchy. Plant clocks also appear to be coupled, yet their coordination centers seem to be more widely distributed than in mammals, operating in a decentralised hierarchy. While detectable heterogeneity between unicellular cyanobacterial clocks indicates a lack of coupling, these instead compensate with their exceptional robustness. Finally, in filamentous cyanobacteria, clocks do appear to be coordinated by direct cell-to-cell coupling.

But why does coupling vary both between and within organisms? And what are the advantages of strongly coupled synchronous clocks or weakly coupled heterogeneous ones? The correlation between clock coupling and multicellularity may hint at functional advantages of circadian coordination in multicellular life. Organization of cells into complex assemblies of specialized tissues and organs is an emergent property of multicellularity. For such systems to function, coordination at every level is important; dysregulation in multicellular organisms is associated with dysfunctional, diseased or aged states (Guseman et al., 2010; Medina and Herranz, 2010; Li et al., 2015; Rosario et al., 2018; Dansereau et al., 2019; Karin et al., 2020; Kruse et al., 2020; Salas-González et al., 2021). With circadian clocks present in most cells of multicellular life, it follows that these would also be coordinated. Indeed, circadian discoordination is similarly linked to detrimental effects in mammals (Li et al., 2013; Nakamura et al., 2015; Kolbe et al., 2019).

Yet between different multicellular organisms and even within individual multicellular organisms, significant differences exist in the strength, distribution and architectures of circadian coupling. For instance, a more centralized hierarchy is present in mammals (Figure 1B) than plants (Figure 4B), with coordination coming from the strongly coupled SCN. These differences may relate to differing physiological mechanisms of entrainment, reflecting fundamental trophic differences between respective kingdoms. For instance, as chemoheterotrophs, nearly all mammalian cells lack intrinsic photosensitivity and thus cannot undergo photoentrainment. Instead, it is vital that the SCN, as a small (order 10,000) collection of cells receiving direct photoreceptive information via the melanopsin-containing retinal ganglion (Berson et al., 2002; Hattar et al., 2002), faithfully entrains to diel light cycles. Subsequently, it must produce highly synchronized outputs, to entrain peripheral oscillators. Stronger coupling between SCN neurons ensures maximal synchrony among this small number of otherwise noisy oscillators (Herzog et al., 2004). SCN coupling also confers robustness to genetic and environmental perturbations, allowing maintenance of SCN-level rhythms even when key clock proteins are mutated or temperatures fluctuate (Liu et al., 2007; Abraham et al., 2018). Yet the coupling is finely tuned; joint experimental and theoretical study shows that the strength of SCN coupling is such that the corresponding oscillator rigidity (increased amplitude and relaxation rate) restricts the range of entrainment to ∼24-hr zeitgeber periods (Abraham et al., 2010). Consequently, the SCN oscillates with a robustness requiring strong zeitgebers, with periods closely matching the intrinsic period, to entrain. Yet coupling is not so strong as to impede entrainment to natural zeitgeber strengths nor adjustment for seasonal variation (Abraham et al., 2010; Hannay et al., 2020). The SCN has therefore been described as “filtering external noise”, ensuring entrainment only occurs to true diel dynamics amidst otherwise fluctuating daily conditions (Abraham et al., 2010). In contrast, peripheral clocks can afford less coupling and weaker oscillators (decreased amplitude and relaxation rate), being subjected to less zeitgeber noise. Mathematical modeling has demonstrated that weak coupling among peripheral tissues increases ranges of entrainment and responsiveness to SCN zeitgebers for achieving synchrony, by maintaining a degree of coherence at all times (Nagoshi et al., 2004; Welsh et al., 2004; Yoo et al., 2004; Rougemont and Naef, 2007; Leise et al., 2012). Furthermore, intrinsic noise and subsequent heterogeneity among weakly coupled clocks may even facilitate oscillations (Leise et al., 2012).

Coupling, by maintaining relative clock synchrony, may also resist the predisposition to cell cycle phase-locking that the TTFL-based clocks of mammals and plants have (Paijmans et al., 2016). This is supported by some evidence showing maintenance of mammalian clock synchronization with dexamethasone treatment prevents 1:1 phase-locking of the clock to cell cycle (Feillet et al., 2014).

Compared to mammals, plants have decentralized, but nonetheless hierarchical circadian systems (Endo et al., 2014; Endo, 2016; Gould et al., 2018; Greenwood et al., 2019). It is now thought that weak coupling exists throughout the plant circadian system, which is further subject to coordination from clocks in the shoot apex and root tip (Figure 4B) (James et al., 2008; Takahashi et al., 2015; Gould et al., 2018; Greenwood et al., 2019). Again, this may relate to the photoautotrophic life for which plants have evolved. Photoreceptors conferring intrinsic photosensitivity and direct entrainability to sunlight rhythms are widely expressed among plant cells. In physiological conditions, the weakly coupled clocks throughout the plant are exposed to and independently synchronize with sunlight, reducing the need for strong synchronizing signals from a single central oscillator. In roots, being relatively hidden from sunlight, photosynthetic signals and light piped via the shoot may facilitate coordination (James et al., 2008; Takahashi et al., 2015; Bordage et al., 2016; Nimmo, 2018). The weak coupling connecting plant clocks, according to the principles outlined by Abraham et al. in 2010, likely imparts lower oscillator rigidity and greater entrainment flexibility (Abraham et al., 2010). Plant clock flexibility was recently emphasized by studies from Bordage et al. (2016) and Greenwood et al. (2019), which revealed coupled clocks in different *Arabidopsis* seedling organs have varied zeitgeber sensitivities, resulting in different intrinsic clock periods, reflecting their diverse roles (Greenwood et al., 2019). Weak coupling was posited to allow flexibility for organ- and tissue-specific timing, according to particular clock inputs and outputs, while maintaining interorgan coordination. In this context, along with other daily physiological processes, it is thought to coordinate important organ-specific changes, including hypocotyl elongation and even modifying the rhizosphere (Nusinow et al., 2011; Hubbard et al., 2018).

In the multicellular filaments of the cyanobacterium *Anabaena* sp. PCC 7210, though clocks are coupled, these do not appear to have a hierarchical structure as in the multicellular eukaryotes discussed (Arbel-Goren et al., 2021). Nonetheless, the fact that clocks are coupled in even such simple multicellular systems highlights the importance of circadian coordination to multicellularity.

Considering how clock coupling pairs well with multicellularity, the observed lack of coupling in unicellular cyanobacteria is perhaps expected (Mihalcescu et al., 2004; Amdaoud et al., 2007). It seems reasonable that unicellular organisms, evolved without reliance upon coordinated multicellular structures, have not invested in clock coupling. Yet, in the absence of homeostatic multicellular structures, unicellular organisms must negotiate greatly fluctuating environmental inputs. Furthermore, the diminutive cell and genome sizes of bacteria, containing fewer genes and biomolecules (Rosenfeld et al., 2005; Milo, 2013; Padovan-Merhar et al., 2015; Walker et al., 2016), result in potentially more replication effect- and noise-prone intracellular environments than eukaryotic cells (Elowitz et al., 2002; Rosenfeld et al., 2005; Süel et al., 2007; Walker et al., 2016; Bertaux et al., 2018). Without coupling, unicellular cyanobacterial clocks instead overcome these obstacles with remarkable robustness and direct heritability of circadian information (Figure 8A) (Mihalcescu et al., 2004). Models of cyanobacterial clock phosphorylation dynamics have highlighted the importance of KaiA sequestration negative feedback in conferring robustness to protein level fluctuations (Clodong et al., 2007). Further theoretical studies argue that cyanobacterial clock robustness also stems from its TTFL-supplemented core PTO, which, along with constant presence of at least 4 (an independently determined number, significant for its consistency with known chromosome numbers (Griese et al., 2011; Jain, Vijayan and O’Shea, 2012), consecutively replicating chromosome copies (Jain, Vijayan and O’Shea, 2012), confers resistance to cell cycle phase-locking (Paijmans et al., 2016).

Clock protein numbers and specific stoichiometries also enhance unicellular cyanobacterial PTO robustness (Chew et al., 2018). Chew et al. investigated this using a clock-tunable *S. elongatus* strain, featuring theophylline-controllable clock protein synthesis. Colony-level bioluminescence and single-cell-level fluorescence assays revealed that high clock protein numbers (10,000s per cell) are key to *S. elongatus* clock maintaining synchrony and robustness to noise. Stochastic simulations of a simplified cyanobacterial PTO also highlighted parts of the molecular clockwork most susceptible to internal noise. In particular, the KaiA-dependent negative feedback loop sustains oscillations but introduces timing error at low KaiA numbers. Chew et al. also explored circadian behavior in the marine cyanobacterium *P. marinus*, which possesses a 10-20-fold lower cell volume than *S. elongatus* and lacks KaiA (Dufresne et al., 2003; Holtzendorff et al., 2008) correspondingly operating as an ‘hourglass’ timer (Schmelling et al., 2017; Chew et al., 2018; Kawamoto et al., 2020). In this species, where clock proteins are maintained at significantly lower numbers (Chew et al., 2018), the clock is susceptible to even greater internal stochasticity. However, in the high-noise, low-protein copy-number environments of these tiny cells, lacking the noise-prone KaiA feedback loop outperforms the full KaiABC clock when synchronizing to highly regular zeitgebers (Chew et al., 2018).

Interestingly, though homologues of all three core cyanobacterial clock genes have been identified in the multicellular filamentous cyanobacterium *Anabaena* sp. PCC 7210, they are not expressed to the same high levels as in *S. elongatus* (Arbel-Goren et al., 2021). It is possible therefore, that the nearest-neighbour coupling identified in this species may compensate for the corresponding decrease in robustness this might cause.

## Unnatural clocks: synthetic biological oscillators

The past two decades have brought synthetic biology from its nascency to the well-established field known today. Shortly after the appearance of fluorescent and bioluminescent reporters in chronobiology, their exploratory use as synthetic biological circuit outputs commenced (Elowitz and Leibler, 2000; Gardner et al., 2000). Perhaps unknown to many synthetic biologists, much motivation for producing certain foundational synthetic biological circuits stemmed from a fascination with circadian oscillators. Synthetic oscillators could provide a controlled, simplified and orthogonal system for investigating the core genetic structures of complex circadian counterparts (Elowitz and Leibler, 2000; Garcia-Ojalvo et al., 2004; Hinze et al., 2011).

Although genetic circuitry has developed over time, incorporating new standardized parts, the underlying principle of synthetic oscillators, as synthetic TTFLs, has remained largely the same. Though far simpler than circadian TTFLs, remarkably synchronous and robust oscillators have been built that might one day match the capabilities of circadian clocks.

**Figure 9:**
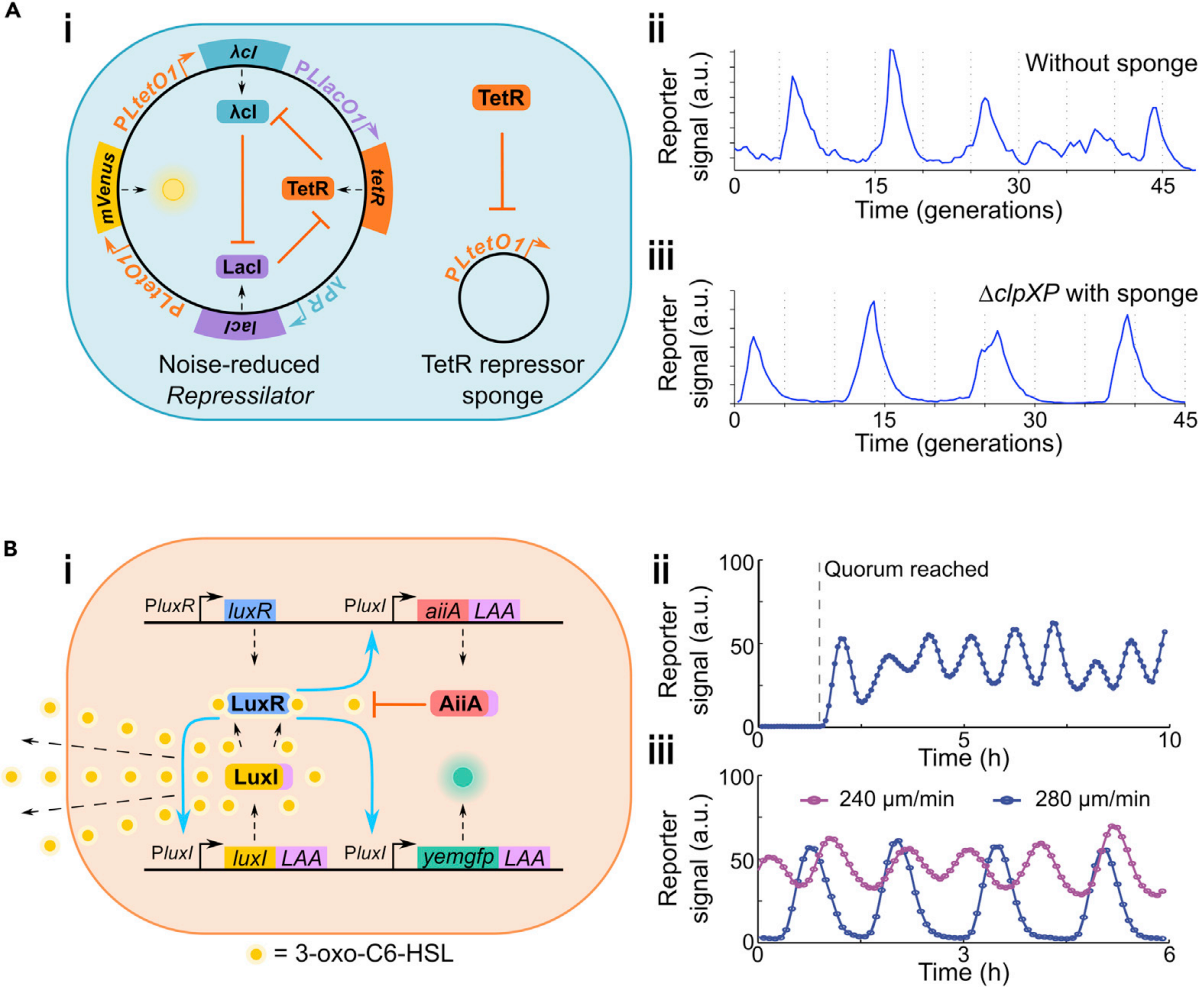
Synthetic clocks (A) The noise-reduced *Repressilator* of Potvin-Trottier et al. (2016). (i) Genetic mechanism. The original *Repressilator* has been optimized in the following ways to make it highly robust within *E. coli*: The reporter has been placed on the same lower noise plasmid as the *Repressilator* circuit; competition for protein degradation has been eliminated by removing all degradation tags and deleting the *clpXP* protease; TetR repressor-binding “sponges” have been introduced to sequester low levels of TetR, eliminating this source of noise; (ii) Fluorescence data of the noisy original *Repressilator*, and (iii) the new robust *Repressilator* (figures adapted, with permission, from Potvin-Trottier, L. et al. (2016) ‘Synchronous long-term oscillations in a synthetic gene circuit’, *Nature*, 538(7626), pp. 514–517. https://doi.org/10.1038/nature19841. Copyright 2016 by Macmillan Publishers Ltd, part of Springer Nature). (B) The quorum-sensing-coupled oscillator of Danino et al. (2010). (i) Genetic mechanism. SsRA-LAA degradation-tagged variants of the 3-oxo-C6-HSL synthase-producing *luxI* and homoserine lactonase-producing *aiiA* genes are placed under the P*luxI* promoter in *E. coli*. In the presence of constitutive LuxR (driven by the P*luxR* promoter), which complexes with 3-oxo-C6-HSL and activates P*luxI* promoters, more 3-oxo-C6-HSL is produced along with the HSL-degrading AiiA. An autoinduction loop with delayed negative feedback thus forms, resulting in an activator-inhibitor oscillator producing robust 3-oxo-C6-HSL oscillations. As an intercellular signaling molecule, 3-oxo-C6-HSL migrates between cells, resulting in each cellular oscillator contributing to and receiving regulation from the resulting oscillations in the shared 3-oxo-C6-HSL pool. Inclusion of a final P*luxI* promoter-driven degradation-tagged YemGFP reporter provides direct readout of 3-oxo-C6-HSL levels. (ii) Fluorescence data taken in a microfluidic device. After ∼1.5 hr quorum is reached and cells begin to oscillate. (iii) Higher flow rates (blue line, 280 μm/min) result in higher peak-to-peak amplitude in 3-oxo-C6-HSL oscillations, reflected in greater amplitude and period oscillations in reporter signal than lower flow rates (magenta line, 240 μm/min) (figures adapted, with permission, from Danino, T. et al. (2010) ‘A synchronized quorum of genetic clocks’, *Nature*, 463(7279), pp. 326–330. https://doi.org/10.1038/nature08753. Copyright 2010 by Macmillan Publishers Limited).

Perhaps the best-known synthetic biological circuit is the *Repressilator* (Elowitz and Leibler, 2000). Comprising a loop of three sequentially inhibitory genetic modules, linked to a fluorescent reporter, it induces remarkable oscillations in single *Escherichia coli* cells. Though noisy and heterogeneous compared to circadian oscillators (Figure 9Aii), it was nonetheless a synthetic biological *tour de force*, paving the way for an abundance of bacterial (Atkinson et al., 2003; Danino et al., 2010; Chen et al., 2015; Potvin-Trottier et al., 2016; Kim et al., 2019; Santos-Moreno et al., 2020), mammalian (Tigges et al., 2009, 2010) and even cell-free oscillators (Niederholtmeyer et al., 2015). For the purposes of this review, we will focus on those that maintain population-level synchrony, akin to circadian clocks.

## Synchrony in synthetic clocks: Noise reduction or coupling?

### Maintaining synchrony by eliminating noise

Exemplified by cyanobacterial clocks, relative synchrony can be maintained among clocks without coupling, by increasing robustness. This principle has been applied to the *Repressilator*; optimized to maintain impressive synchrony without coupling, by reducing stochastic fluctuation at multiple levels (Potvin-Trottier et al., 2016). First, stochasticity in fluorescent reporting has been reduced by placing it on the same lower noise plasmid as the *Repressilator* circuit. Second, by removing reporter and *Repressilator* component degradation competition has been reduced between the two, while also permitting higher protein copy numbers. Finally, after identifying oversensitivity of TetR-responsive modules to be the circuit’s largest noise contributor, TetR repressor-binding “sponges” have been introduced, eliminating this noise through sequestration of low-level TetR (Figure 9Ai). In combination, these changes have reduced noise in the original *Repressilator* (Figure 9Aii) such that population-level synchrony is maintained over tens of generations (Figure 9Aiii) (Potvin-Trottier et al., 2016). Most recently a robust CRISPR-interference-based *Repressilator*, the *CRISPRlator*, has been built (Santos-Moreno et al., 2020). Reducing variation by placing all sequentially repressive sgRNA modules on the same plasmid and relying on a constant, shared cellular pool of dCas9 and Csy4 RNase, has allowed impressively synchronous, heritable 3-colour oscillations, without coupling (Santos-Moreno et al., 2020).

### Maintaining synchrony through coupling

As evident from multicellular circadian systems, mutual synchrony can be achieved with noisy oscillators through coupling. Indeed, theoretical study has long predicted populations of oscillators, when coupled, might transition to synchronous states (Winfree, 1967).

Coupling between synthetic oscillators has primarily involved quorum-sensing machinery from Gram-negative bacteria. These quorum-sensing systems are intercellular coordination mechanisms for controlling population-gated behaviors. They are characterized by three components: homoserine lactone intercellular autoinducer (HSL) synthases (“I” proteins), receiver proteins (“R” proteins) and quorum-sensing promoters, which are activated by cognate HSL:receiver protein complexes. A well-characterized example is the Lux quorum-sensing system from the bioluminescent marine bacterium *Vibrio fischeri*, consisting of LuxI, LuxR and P*luxI* (Figure 9B). Theoretical (McMillen et al., 2002; Garcia-Ojalvo et al., 2004) and experimental (Fernández-Niño et al., 2017) implementation of this system has suggested it could be used to couple and mutually synchronize *Repressilators*.

The Lux quorum-sensing system has itself been used as the base oscillatory unit of perhaps the best-known quorum-sensing-coupled oscillator, published by Danino et al. (2010). Comprising three P*luxI*-driven modules controlling quorum-sensing machinery from *V. fischeri* and *Bacillus thuringiensis*, alongside a YemGFP reporter in *E. coli*, the oscillator employs an architecture featuring positive and negative regulation. An activator (in this case a homoserine lactone (HSL)) activates itself as well as its inhibitor (in this case a homoserine lactonase, AiiA) forming a dual-feedback, activator-inhibitor oscillator (Figure 9Bi). Within microfluidic devices, oscillator populations could demonstrate impressively synchronous oscillations (Figure 9Bii) and spatiotemporal waves (Danino et al., 2010). Modifications upon this oscillator have since demonstrated large-scale inter-colony synchronization in arrays of microfluidic ‘biopixels’ (Prindle et al., 2011) and even the frequency-encoding of measurement information (Prindle et al., 2014).

More recently, researchers have made use of the orthogonal Rhl and Cin quorum-sensing systems from *Pseudomonas aeruginosa* and *Rhizobium leguminosarum*. Publications from Chen et al. in 2015 and Kim et al. in 2019 describe a novel *E. coli* co-culture oscillator utilizing these components (Chen et al., 2015; Kim et al., 2019). This approach houses orthogonal activation and repression components, as well as spectrally distinct fluorescent reporters, in separate strains. Within an extended microfluidic device, this oscillator has also demonstrated strikingly synchronous fluorescence oscillations and interesting spatiotemporal dynamics (Chen et al., 2015; Kim et al., 2019).

Mathematical modeling has been key in informing the above genetic circuits designs. Unlike circadian clocks, where complex TTFLs interact in ways not yet entirely known, synthetic biologists can hand-pick characterized components to use in small oscillatory networks, greatly facilitating model development. The core eukaryotic clock networks are thought to consist of tens of genes (Ukai and Ueda, 2010; Nohales and Kay, 2016), while simple synthetic oscillators may comprise an order of magnitude fewer (Elowitz and Leibler, 2000). Thus, while circadian clocks are generally, for simplicity, modeled as generic coupled oscillators (Liu et al., 1997; Achermann and Kunz, 1999; Beck et al., 2001; Mihalcescu et al., 2004; Amdaoud et al., 2007; Rougemont and Naef, 2007; Gu et al., 2015, 2016; Gould et al., 2018; Myung et al., 2018; Schmal et al., 2018; Greenwood et al., 2019; Hannay et al., 2020), synthetic biological oscillators are more readily modeled with systems of differential equations describing the transcription, translation, degradation and interaction of each species (Elowitz and Leibler, 2000; Danino et al., 2010; Chen et al., 2015; Kim et al., 2019; Santos-Moreno et al., 2020).

## Oscillator structure in circadian and synthetic clocks

Considering an initial motivation for building synthetic oscillators was to recreate simplified clock TTFLs, one might think known clock networks informed their design. However, when these were first designed, such information was less known, thus, they served more as proofs-of-principle for *potential* mechanisms underpinning circadian clocks. Better understanding of clock networks now allows the structures and resultant properties of circadian and synthetic clocks to be compared.

**Figure 10:**
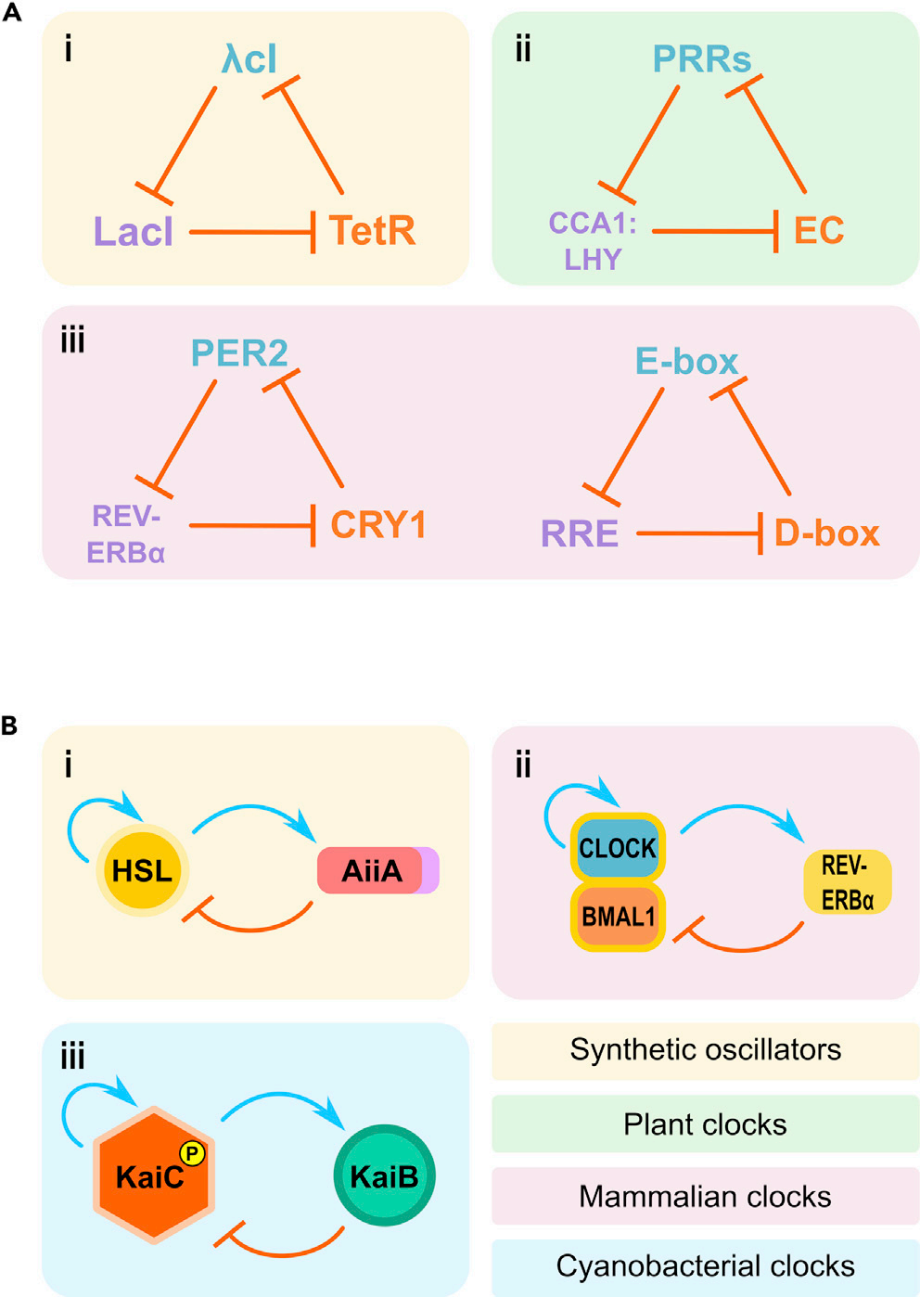
Shared circadian and synthetic oscillator network motifs (A) *Repressilator* motifs. (i) The synthetic biological *Repressilator* (Elowitz and Leibler, 2000), (ii) the plant clock core *Repressilator* (Pokhilko et al., 2012), (iii) two mammalian clock core *Repressilators* (Ukai-Tadenuma et al., 2011; Pett et al., 2016). (B) Activator-inhibitor oscillator motifs. (i) The synthetic biological quorum-sensing oscillator from Danino et al. (2010); (ii) the mammalian clock core TTFL activator-inhibitor motif (Wu et al., 2017); (iii) The cyanobacterial clock core PTO activator-inhibitor motif features phosphorylated KaiC enhancing its own expression and that of KaiB, which negatively feeds back by promoting KaiC dephosphorylation (Wu et al., 2017). Blue pointed arrows and orange flat-headed arrows signify positive and negative feedback, respectively.

The *Repressilator* (Figure 10Ai), when coupled, has long been proposed for use in modeling simplified clock networks (Hinze et al., 2011). Significantly, the *Repressilator* also repeatedly emerges as a core motif from analyses of real circadian clock networks (Ukai-Tadenuma et al., 2011; Pokhilko et al., 2012; Pett et al., 2016; Wu et al., 2017). In 2011, Ukai-Tadenuma et al., using a minimal-network representation of the mammalian clock, found delayed negative feedback and *Repressilator* motifs at its core. This clock *Repressilator* featured E-box-containing genes inhibiting RRE-containing genes, inhibiting D-box-containing genes, inhibiting E-box-containing genes (Figure 10Aiii) (Ukai-Tadenuma et al., 2011). Studies using alternative methods also report *Repressilator* motifs at the core of the mammalian clock (Pett et al., 2016). Pett et al., through deeper analysis of a previous data-driven delay differential equation clock model (Korenčič et al., 2014), identified a shortlist of 17 key regulatory interactors in the mammalian clock. By applying a combinatorial clamping method, these could be distilled down to a robustly oscillating, sequentially inhibitory loop, a *Repressilator*, comprising *Cry1* inhibiting *Per2*, inhibiting *Rev-erbα*, inhibiting *Cry1* (Figure 10Aiii). Interestingly, it is thought *Repressilators* and other motifs common to synthetic biology may be particularly amenable to the fundamental clock property of temperature-compensated oscillations (Wu et al., 2017). Wu et al. randomly assigned 10,000–100,000 parameter sets to 2423 independent coarse-grained networks containing 2 or 3 nodes. Represented by Arrhenius equation-modulated (introducing temperature effects) coupled ordinary differential equations, four simple motifs were identified, which in combination, could make up temperature-compensated clock networks (Wu et al., 2017). One core motif most readily capable of oscillating was again, a *Repressilator*, citing the clock loop proposed by Ukai-Tadenuma and others in 2011 as a potential mechanism (Ukai-Tadenuma et al., 2011). Additionally, other core motifs associated with temperature-compensated oscillations emerged from the analysis: delayed negative feedback and activator-inhibitor motifs (Wu et al., 2017). Variants of these have been used successfully in a variety of synthetic biological circuits (Figure 10Bi), yet also appear in mammalian (Figure 10Biii) and cyanobacterial clocks (Figure 10Biii), further demonstrating parallels between evolved and synthetic clock networks (Atkinson et al., 2003; Stricker et al., 2008; Danino et al., 2010; Chen et al., 2015; Kim et al., 2019).

*Repressilator* motifs are not unique to mammalian clocks. Integrating new information on the *Arabidopsis*‘Evening Complex’ (EC) to restructure a mathematical clock network model, a 2012 report from Pokhilko et al. revealed a *Repressilator* to be central to the plant clock network (Pokhilko et al., 2012). Comprising the EC inhibiting *PRR* genes, inhibiting *CCA1*:*LHY*, inhibiting the EC, this suggests such motifs might be pervasive among eukaryotic clocks (Figure 10Aii).

Though *Repressilators* do appear to be core motifs in these clocks, the importance of supplementary positive feedback loops should not be understated. Indeed, it is thought that oscillators comprising both positive and negative feedback possess greater robustness and tunability than those comprising purely negative feedback, such as the *Repressilator* or even the widely used *Goodwin* oscillator (Tsai et al., 2008). Nevertheless, such coarse-grained models have effectively recapitulated fundamental clock properties, including self-sustained oscillations, entrainment and temperature compensation (Ruoff and Rensing, 1996; Achermann and Kunz, 1999; Kunz and Achermann, 2003; Gonze et al., 2005; Bernard et al., 2007; To et al., 2007; Hafner et al., 2012). It appears therefore, that evolution and synthetic biologists have converged upon similar motifs to generate effective oscillators. Yet, perhaps owing to their comparative simplicity, besides replicating oscillations, there are limited reports of other core clock characteristics being recreated by synthetic biological oscillators. One important clock property that is yet to be explicitly demonstrated with synthetic biological oscillators is that of growth rate-independent timekeeping. However, some examples do exist for other properties. These include Mondragón-Palomino et al.’s description of an Arabinose- and IPTG-entrainable synthetic oscillator in 2011 (Mondragón-Palomino et al., 2011), as well as Hussain et al. demonstrating an impressive temperature-compensated synthetic oscillator shortly after (Hussain et al., 2014).

Parallels can also be drawn between circadian and synthetic clocks in their mechanisms for maintaining inter-oscillator synchrony and robustness. Most notable is inter-oscillator coupling: in both synthetic biological and circadian systems, diffusible molecule-mediated coupling effectively synchronizes oscillators. In synthetic biological oscillators, this predominantly takes the form of HSLs, coupling oscillators via quorum-sensing machinery (McMillen et al., 2002; Garcia-Ojalvo et al., 2004; Danino et al., 2010; Chen et al., 2015; Kim et al., 2019). Though surprisingly little is known about what mediates circadian coupling, studies also imply diffusible molecule involvement, at least in mammals. For example, VIP, in addition to its role along with AVP and GABA in coordinating SCN subcompartments via synaptic communication (Albus et al., 2005; Maywood et al., 2006), also transmits paracrine signals. Maywood et al. demonstrated this in 2011, through genotype-specific circadian rhythm restoration in synaptically disconnected VIP-null SCN slices by VIP+ SCN slices (Maywood et al., 2011). Experimental and theoretical studies also suggest unknown paracrine signals likely synchronize peripheral oscillators to the SCN and potentially weakly couple them to each other (Nagoshi et al., 2004; Welsh et al., 2004; Rougemont and Naef, 2007; Noguchi et al., 2013). Additionally, diffusible factors in the cerebrospinal fluid are suspected to mediate CP-to-SCN coupling (Myung et al., 2018).

Reflecting the complexity and breadth of circadian systems, there are also synchronization mechanisms that have not, or cannot yet be exploited in synthetic biology. For example, a key coupling mediator in the SCN is thought to be electrochemical signaling between neurons, as evidenced by the desynchronizing effects of the action potential inhibitor TTX (Yamaguchi et al., 2003; Webb et al., 2009; Abel et al., 2016; Taylor et al., 2017). Additionally, though recent reports indicate SCN clocks are not coupled through gap junctions (Diemer et al., 2017), they are nonetheless implicated in the coupling between CP clocks (Myung et al., 2018). Furthermore, evidence suggests the clocks of plants and filamentous cyanobacteria are also coupled through direct cytoplasmic connections, via their analogous plasmodesmata and septal junctions, respectively (Takahashi et al., 2015; Arbel-Goren et al., 2021).

Parallels also exist in coupling-free strategies for maintaining oscillator synchrony. Studies suggest both fibroblast and cyanobacterial cells pass circadian timing between generations to remain synchronous (Mihalcescu et al., 2004; Nagoshi et al., 2004; Arbel-Goren et al., 2021). Similarly, optimized *Repressilator* designs have shown periods of 14–17 generations, during which oscillator phase is passed to daughter cells with only slight intergenerational shifting (Potvin-Trottier et al., 2016; Santos-Moreno et al., 2020). These optimized *Repressilators* are also noteworthy for their robustness, maintaining synchrony without coupling, through eliminating sources of noise in the oscillator network. Cyanobacterial clocks have also demonstrated robustness and synchrony without coupling by reducing component susceptibility to internal and external noise. This occurs through negative feedback KaiA sequestration and expression of clock proteins in large numbers (Clodong et al., 2007; Chew et al., 2018). The theoretical study also highlights the applicability of cyanobacterial circadian clock design in synthetic oscillators, specifically in retaining robustness to- and independence from cell cycle (Paijmans et al., 2016; Paijmans et al., 2017). This places particular emphasis on the constant presence of multiple, consecutively replicating chromosome copies to avoid cell cycle phase-locking. This can be expanded to the *Repressilator* (Elowitz and Leibler, 2000) and original synthetic dual feedback oscillator (Stricker et al., 2008), where, akin to Chew et al.s’ 2018 observations of the cyanobacterial clock (Chew et al., 2018), higher oscillator component copy numbers improve oscillator robustness (Potvin-Trottier et al., 2016; Paijmans et al., 2017).

## Conclusions and future perspectives

This review has explored theoretical and experimental studies of cell-to-cell coupling in three main circadian clock models. From these we have learnt that across the kingdoms of life, varying degrees of coupling exist. Multicellular life has evolved to operate in a fine balance: limited intrinsic noise with weaker coupling confers ability to maintain oscillations and flexibility to synchronize, entraining directly, or via a central pacemaker to the diel dynamics of the world (Leise et al., 2012; Webb et al., 2012; Gu et al., 2015, 2016; St John and Doyle, 2015; Gould et al., 2018; Greenwood et al., 2019); stronger coupling ensures robustness to perturbation and sustained oscillations, coordinating individual cells within complex multicellular ensembles (Pando et al., 2002; Liu et al., 2007; Rougemont and Naef, 2007; Abraham et al., 2018). In unicellular organisms, where there is no evidence for significant coupling, clocks have instead evolved impressive robustness, optimized for noisy cellular environments (Clodong et al., 2007; Paijmans et al., 2016; Chew et al., 2018).

Much of our understanding has been facilitated by advances in single-cell techniques. Combined with the latest modeling methods, studies have shown relative coupling strength to be quantifiable by observing the distributions of three key properties: period, phase and amplitude, in single-cell circadian data (Schmal et al., 2018). Future efforts should now utilize these theoretical advances in large-scale comparative studies of period, phase and amplitude distributions for all types of circadian oscillator. This will prove extremely useful in facilitating the meaningful comparison of relative coupling strengths between circadian clocks in different tissues and organisms across the Tree of Life. In addition, efforts should concentrate in further elucidating the specific agents that mediate the clock coupling in these different systems.

We have also consolidated the fields of chronobiology and synthetic biology, comparing design principles and properties of circadian and synthetic clocks. Evolution and synthetic biologists have converged upon the same strategies to maintain oscillator synchrony: utilizing cell-to-cell coupling (Danino et al., 2010; Prindle et al., 2011, 2014; Chen et al., 2015; Kim et al., 2019) or extreme robustness without coupling (Potvin-Trottier et al., 2016; Santos-Moreno et al., 2020). Furthermore, data-driven clock models have revealed circadian and synthetic oscillators share core motifs, including delayed negative feedback, activator-inhibitor and the *Repressilator* (Ukai-Tadenuma et al., 2011; Pokhilko et al., 2012; Pett et al., 2016; Wu et al., 2017). Importantly, these motifs have been highlighted for their role in replicating key oscillatory and temperature-compensated clock properties. With this in mind, future efforts should continue these developments, exploring the extent to which synthetic biological systems can capture more complex clock behaviors, to test our understanding of circadian clock design principles. These might also be exploited in biotechnological applications, for example in the optimal timing and coordination of heterologous genetic circuits for metabolic engineering or even therapeutic uses. Thus, as life on Earth has benefited from the coordination of circadian clocks, perhaps future life may also benefit from synthetic coordination, as we close the gap between circadian and synthetic clocks.

## References

[bib1] Abel J.H., Meeker K., Granados-Fuentes D., St John P.C., Wang T.J., Bales B.B., Doyle F.J., Herzog E.D., Petzold L.R. (2016). Functional network inference of the suprachiasmatic nucleus. Proc. Natl. Acad. Sci. U. S. A..

[bib2] Abraham U., Granada A.E., Westermark P.O., Heine M., Kramer A., Herzel H. (2010). Coupling governs entrainment range of circadian clocks. Mol. Syst. Biol..

[bib3] Abraham U., Schlichting J.K., Kramer A., Herzel H. (2018). Quantitative analysis of circadian single cell oscillations in response to temperature. PLoS ONE.

[bib4] Achermann P., Kunz H. (1999). Modeling circadian rhythm generation in the suprachiasmatic nucleus with locally coupled self-sustained oscillators: phase shifts and phase response curves. J. Biol. Rhythms.

[bib5] Adams S., Manfield I., Stockley P., Carré I.A. (2015). Revised morning loops of the arabidopsis circadian clock based on analyses of direct regulatory interactions. PLoS One.

[bib6] Akashi M., Takumi T. (2005). The orphan nuclear receptor RORα regulates circadian transcription of the mammalian core-clock Bmal1. Nat. Struct. Mol. Biol..

[bib7] Albus H., Vansteensel M.J., Michel S., Block G.D., Meijer J.H. (2005). A GABAergic mechanism is necessary for coupling dissociable ventral and dorsal regional oscillators within the circadian clock. Curr. Biol..

[bib8] Amdaoud M., Vallade M., Weiss-Schaber C., Mihalcescu I. (2007). Cyanobacterial clock, a stable phase oscillator with negligible intercellular coupling. Proc. Natl. Acad. Sci. U. S. A..

[bib9] Andersen J.B., Sternberg C., Poulsen L.K., Bjorn S.P., Givskov M., Molin S. (1998). New unstable variants of green fluorescent protein for studies of transient gene expression in bacteria. Appl. Environ. Microbiol..

[bib10] Arbel-Goren R., Buonfiglio V., Di Patti F., Camargo S., Valladares A., Flores E., Herrero A., Fanelli D., Stavans J. (2021). Robust, coherent and synchronized circadian clock-controlled oscillations along Anabaena filaments. eLife.

[bib11] Atkinson M.R., Savageau M.A., Myers J.T., Ninfa A.J. (2003). Development of genetic circuitry exhibiting toggle switch or oscillatory behavior in *Escherichia coli*. Cell.

[bib12] Balsalobre A., Damiola F., Schibler U. (1998). A serum shock induces circadian gene expression in mammalian tissue culture cells. Cell.

[bib13] Bargiello T.A., Jackson F.R., Young M.W. (1984). Restoration of circadian behavioural rhythms by gene transfer in Drosophila. Nature.

[bib14] Beck F., Blasius B., Lüttge U., Neff R., Rascher U. (2001). Stochastic noise interferes coherently with a model biological clock and produces specific dynamic behaviour. Proc. Biol. Sci..

[bib15] Bernard S., Gonze D., Cajavec B., Herzel H., Kramer A. (2007). Synchronization-induced rhythmicity of circadian oscillators in the suprachiasmatic nucleus. PLoS Comput. Biol..

[bib16] Berson D.M., Dunn F.A., Takao M. (2002). Phototransduction by retinal ganglion cells that set the circadian clock. Science.

[bib17] Bertaux F., Marguerat S., Shahrezaei V. (2018). Division rate, cell size and proteome allocation: impact on gene expression noise and implications for the dynamics of genetic circuits. R. Soc. Open Sci..

[bib18] Bognár L.K., Hall A., Adám E., Thain S.C., Nagy F., Millar A.J. (1999). The circadian clock controls the expression pattern of the circadian input photoreceptor, phytochrome B. Proc. Natl. Acad. Sci. U. S. A..

[bib19] Bordage S., Sullivan S., Laird J., Millar A.J., Nimmo H.G. (2016). Organ specificity in the plant circadian system is explained by different light inputs to the shoot and root clocks. New Phytol.

[bib20] Bray M.S., Shaw C.A., Moore M.W.S., Garcia R.A.P., Zanquetta M.M., Durgan D.J., Jeong W.J., Tsai J.-Y., Bugger H., Zhang D. (2008). Disruption of the circadian clock within the cardiomyocyte influences myocardial contractile function, metabolism, and gene expression. Am. J. Physiol. Heart Circ. Physiol..

[bib21] Chabot J.R., Pedraza J.M., Luitel P., van Oudenaarden A. (2007). Stochastic gene expression out-of-steady-state in the cyanobacterial circadian clock. Nature.

[bib22] Chang Y.-G., Cohen S.E., Phong C., Myers W.K., Kim Y.-I., Tseng R., Lin J., Zhang L., Boyd J.S., Lee Y. (2015). Circadian rhythms. A protein fold switch joins the circadian oscillator to clock output in cyanobacteria. Science.

[bib23] Chang Y.-G., Tseng R., Kuo N.-W., LiWang A. (2012). Rhythmic ring-ring stacking drives the circadian oscillator clockwise. Proc. Natl. Acad. Sci. U. S. A..

[bib24] Chen W.W., Takahashi N., Hirata Y., Ronald J., Porco S., Davis S.J., Nusinow D.A., Kay S.A., Mas P. (2020). A mobile ELF4 delivers circadian temperature information from shoots to roots. Nat Plants.

[bib25] Chen Y., Kim J.K., Hirning A.J., Josić K., Bennett M.R. (2015). Synthetic biology. Emergent genetic oscillations in a synthetic microbial consortium. Science.

[bib26] Chew J., Leypunskiy E., Lin J., Murugan A., Rust M.J. (2018). High protein copy number is required to suppress stochasticity in the cyanobacterial circadian clock. Nat. Commun..

[bib27] Chow B.Y., Helfer A., Nusinow D.A., Kay S.A. (2012). ELF3 recruitment to the PRR9 promoter requires other Evening Complex members in the Arabidopsis circadian clock. Plant Signal. Behav..

[bib28] Clodong S., Dühring U., Kronk L., Wilde A., Axmann I., Herzel H., Kollmann M. (2007). Functioning and robustness of a bacterial circadian clock. Mol. Syst. Biol..

[bib29] Cohen S.E., Golden S.S. (2015). Circadian rhythms in cyanobacteria. Microbiol. Mol. Biol. Rev..

[bib30] Dai S., Wei X., Pei L., Thompson R.L., Liu Y., Heard J.E., Ruff T.G., Beachy R.N. (2011). Brother of lux arrhythmo is a component of the Arabidopsis circadian clock. Plant Cell.

[bib31] Damiola F., Le Minh N., Preitner N., Kornmann B., Fleury-Olela F., Schibler U. (2000). Restricted feeding uncouples circadian oscillators in peripheral tissues from the central pacemaker in the suprachiasmatic nucleus. Genes Dev..

[bib32] Danino T., Mondragón-Palomino O., Tsimring L., Hasty J. (2010). A synchronized quorum of genetic clocks. Nature.

[bib33] Dansereau G., Wey T.W., Legault V., Brunet M.A., Kemnitz J.W., Ferrucci L., Cohen A.A. (2019). Conservation of physiological dysregulation signatures of aging across primates. Aging Cell.

[bib34] Diemer T., Landgraf D., Noguchi T., Pan H., Moreno J.L., Welsh D.K. (2017). Cellular circadian oscillators in the suprachiasmatic nucleus remain coupled in the absence of connexin-36. Neuroscience.

[bib35] Dowson-Day M.J., Millar A.J. (1999). Circadian dysfunction causes aberrant hypocotyl elongation patterns in Arabidopsis. Plant J.

[bib36] Doyle M.R., Davis S.J., Bastow R.M., McWatters H.G., Kozma-Bognár L., Nagy F., Millar A.J., Amasino R.M. (2002). The ELF4 gene controls circadian rhythms and flowering time in Arabidopsis thaliana. Nature.

[bib37] Duanis-Assaf D., Steinberg D., Chai Y., Shemesh M. (2015). The LuxS based quorum sensing governs lactose induced biofilm formation by Bacillus subtilis. Front. Microbiol..

[bib38] Dufresne A., Salanoubat M., Partensky F., Artiguenave F., Axmann I.M., Barbe V., Duprat S., Galperin M.Y., Koonin E.V., Le Gall F. (2003). Genome sequence of the cyanobacterium Prochlorococcus marinus SS120, a nearly minimal oxyphototrophic genome. Proc. Natl. Acad. Sci. U. S. A..

[bib39] Eelderink-Chen Z., Bosman J., Sartor F., Dodd A.N., Kovács Á.T., Merrow M. (2021). A circadian clock in a nonphotosynthetic prokaryote. Science Advances.

[bib40] Elowitz M.B., Leibler S. (2000). A synthetic oscillatory network of transcriptional regulators. Nature.

[bib41] Elowitz M.B., Levine A.J., Siggia E.D., Swain P.S. (2002). Stochastic gene expression in a single cell. Science.

[bib42] Endo M. (2016). Tissue-specific circadian clocks in plants. Curr. Opin. Plant Biol..

[bib43] Endo M., Shimizu H., Nohales M.A., Araki T., Kay S.A. (2014). Tissue-specific clocks in Arabidopsis show asymmetric coupling. Nature.

[bib44] Ermentrout G.B., Kopell N. (1991). Multiple pulse interactions and averaging in systems of coupled neural oscillators. J. Math. Biol..

[bib45] Farinas B., Mas P. (2011). Functional implication of the MYB transcription factor RVE8/LCL5 in the circadian control of histone acetylation: Role of RVE8/LCL5 in the circadian clock. Plant J.

[bib46] Feillet C., Krusche P., Tamanini F., Janssens R.C., Downey M.J., Martin P., Teboul M., Saito S., Lévi F.A., Bretschneider T. (2014). Phase locking and multiple oscillating attractors for the coupled mammalian clock and cell cycle. Proc. Natl. Acad. Sci. U. S. A..

[bib47] Fernández-Niño M., Giraldo D., Gomez-Porras J.L., Dreyer I., González Barrios A.F., Arevalo-Ferro C. (2017). A synthetic multi-cellular network of coupled self-sustained oscillators. PLoS ONE.

[bib48] Fukuda H., Nakamichi N., Hisatsune M., Murase H., Mizuno T. (2007). Synchronization of plant circadian oscillators with a phase delay effect of the vein network. Phys. Rev. Lett..

[bib49] Fukuda H., Ukai K., Oyama T. (2012). Self-arrangement of cellular circadian rhythms through phase-resetting in plant roots. Phys. Rev. E Stat. Nonlin. Soft Matter Phys..

[bib50] Gaddameedhi S., Selby C.P., Kaufmann W.K., Smart R.C., Sancar A. (2011). Control of skin cancer by the circadian rhythm. Proc. Natl. Acad. Sci. U. S. A..

[bib51] Garcia-Ojalvo J., Elowitz M.B., Strogatz S.H. (2004). Modeling a synthetic multicellular clock: repressilators coupled by quorum sensing. Proc. Natl. Acad. Sci. U. S. A..

[bib52] Gardner T.S., Cantor C.R., Collins J.J. (2000). Construction of a genetic toggle switch in Escherichia coli. Nature.

[bib53] Gekakis N., Staknis D., Nguyen H.B., Davis F.C., Wilsbacher L.D., King D.P., Takahashi J.S., Weitz C.J. (1998). Role of the CLOCK protein in the mammalian circadian mechanism. Science.

[bib54] Gillespie D.T. (1977). Exact stochastic simulation of coupled chemical reactions. J. Phys. Chem..

[bib55] Glass L., Mackey M.C. (1988).

[bib56] Gonze D., Bernard S., Waltermann C., Kramer A., Herzel H. (2005). Spontaneous synchronization of coupled circadian oscillators. Biophys. J..

[bib57] Goodwin B.C. (1965). Oscillatory behavior in enzymatic control processes. Adv. Enzyme Regul..

[bib58] Gould P.D., Domijan M., Greenwood M., Tokuda I.T., Rees H., Kozma-Bognar L., Hall A.J., Locke J.C. (2018). Coordination of robust single cell rhythms in the Arabidopsis circadian clock via spatial waves of gene expression. eLife.

[bib59] Greenwood M., Domijan M., Gould P.D., Hall A.J.W., Locke J.C.W. (2019). Coordinated circadian timing through the integration of local inputs in Arabidopsis thaliana. PLoS Biol.

[bib60] Griese M., Lange C., Soppa J. (2011). Ploidy in cyanobacteria. FEMS Microbiol. Lett..

[bib61] Gu C., Liang X., Yang H., Rohling J.H.T. (2016). Heterogeneity induces rhythms of weakly coupled circadian neurons. Sci. Rep..

[bib62] Gu C., Xu J., Rohling J., Yang H., Liu Z. (2015). Noise Induces Oscillation and Synchronization of the Circadian Neurons. PLoS ONE.

[bib63] Guenthner C.J., Luitje M.E., Pyle L.A., Molyneux P.C., Yu J.K., Li A.S., Leise T.L., Harrington M.E. (2014). Circadian rhythms of PER2::LUC in individual primary mouse hepatocytes and cultures. PLoS One.

[bib64] Guerriero M.L., Pokhilko A., Fernández A.P., Halliday K.J., Millar A.J., Hillston J. (2012). Stochastic properties of the plant circadian clock. J. R. Soc. Interface.

[bib65] Guillaumond F., Dardente H., Giguère V., Cermakian N. (2005). Differential control of Bmal1 circadian transcription by REV-ERB and ROR nuclear receptors. J. Biol. Rhythms.

[bib66] Guseman J.M., Lee J.S., Bogenschutz N.L., Peterson K.M., Virata R.E., Xie B., Kanaoka M.M., Hong Z., Torii K.U. (2010). Dysregulation of cell-to-cell connectivity and stomatal patterning by loss-of-function mutation in Arabidopsis CHORUS (GLUCAN SYNTHASE-LIKE 8). Development.

[bib67] Hafner M., Koeppl H., Gonze D. (2012). Effect of network architecture on synchronization and entrainment properties of the circadian oscillations in the suprachiasmatic nucleus. PLoS Comput. Biol..

[bib68] Hannay K.M., Forger D.B., Booth V. (2020). Seasonality and light phase-resetting in the mammalian circadian rhythm. Sci. Rep..

[bib69] Hannay K.M., Forger D.B., Booth V. (2018). Macroscopic models for networks of coupled biological oscillators. Sci Adv.

[bib70] Harmer S.L., Hogenesch J.B., Straume M., Chang H.S., Han B., Zhu T., Wang X., Kreps J.A., Kay S.A. (2000). Orchestrated transcription of key pathways in Arabidopsis by the circadian clock. Science.

[bib71] Hattar S., Liao H.W., Takao M., Berson D.M., Yau K.W. (2002). Melanopsin-containing retinal ganglion cells: architecture, projections, and intrinsic photosensitivity. Science.

[bib72] Herzog E.D., Aton S.J., Numano R., Sakaki Y., Tei H. (2004). Temporal precision in the mammalian circadian system: a reliable clock from less reliable neurons. J. Biol. Rhythms.

[bib73] Hinze T., Schumann M., Bodenstein C., Heiland I., Schuster S. (2011). Biochemical frequency control by synchronisation of coupled repressilators: an in silico study of modules for circadian clock systems. Comput. Intell. Neurosci..

[bib74] Hirota T., Lee J.W., St John P.C., Sawa M., Iwaisako K., Noguchi T., Pongsawakul P.Y., Sonntag T., Welsh D.K., Brenner D.A. (2012). Identification of small molecule activators of cryptochrome. Science.

[bib75] Hitomi K., Oyama T., Han S., Arvai A.S., Getzoff E.D. (2005). Tetrameric architecture of the circadian clock protein KaiB. A novel interface for intermolecular interactions and its impact on the circadian rhythm. J. Biol. Chem..

[bib76] Holtzendorff J., Partensky F., Mella D., Lennon J.-F., Hess W.R., Garczarek L. (2008). Genome streamlining results in loss of robustness of the circadian clock in the marine cyanobacterium Prochlorococcus marinus PCC 9511. J. Biol. Rhythms.

[bib77] Honma S., Shirakawa T., Katsuno Y., Namihira M., Honma K. (1998). Circadian periods of single suprachiasmatic neurons in rats. Neurosci. Lett..

[bib78] Hsu P.Y., Devisetty U.K., Harmer S.L. (2013). Accurate timekeeping is controlled by a cycling activator in Arabidopsis. eLife.

[bib79] Huang W., Pérez-García P., Pokhilko A., Millar A.J., Antoshechkin I., Riechmann J.L., Mas P. (2012). Mapping the core of the Arabidopsis circadian clock defines the network structure of the oscillator. Science.

[bib80] Hubbard C.J., Brock M.T., van Diepen L.T., Maignien L., Ewers B.E., Weinig C. (2018). The plant circadian clock influences rhizosphere community structure and function. ISME J.

[bib81] Huh I., Zeng J., Park T., Yi S.V. (2013). DNA methylation and transcriptional noise. Epigenetics Chromatin.

[bib82] Hussain F., Gupta C., Hirning A.J., Ott W., Matthews K.S., Josic K., Bennett M.R. (2014). Engineered temperature compensation in a synthetic genetic clock. Proc. Natl. Acad. Sci. U. S. A..

[bib83] Inouye S.-I.T., Kawamura H. (1979). Persistence of circadian rhythmicity in a mammalian hypothalamic “Island” containing the suprachiasmatic nucleus. Proc. Natl. Acad. Sci. U. S. A..

[bib84] Ishiura M., Kutsuna S., Aoki S., Iwasaki H., Andersson C.R., Tanabe A., Golden S.S., Johnson C.H., Kondo T. (1998). Expression of a gene cluster kaiABC as a circadian feedback process in cyanobacteria. Science.

[bib85] Ito H., Kageyama H., Mutsuda M., Nakajima M., Oyama T., Kondo T. (2007). Autonomous synchronization of the circadian KaiC phosphorylation rhythm. Nat. Struct. Mol. Biol..

[bib86] Jain I.H., Vijayan V., O’Shea E.K. (2012). Spatial ordering of chromosomes enhances the fidelity of chromosome partitioning in cyanobacteria. Proc. Natl. Acad. Sci. U. S. A..

[bib87] James A.B., Monreal J.A., Nimmo G.A., Kelly C.L., Herzyk P., Jenkins G.I., Nimmo H.G. (2008). The circadian clock in Arabidopsis roots is a simplified slave version of the clock in shoots. Science.

[bib88] Kamioka M., Takao S., Suzuki T., Taki K., Higashiyama T., Kinoshita T., Nakamichi N. (2016). Direct Repression of Evening Genes by CIRCADIAN CLOCK-ASSOCIATED1 in the Arabidopsis Circadian Clock. Plant Cell.

[bib89] Karin O., Raz M., Tendler A., Bar A., Korem Kohanim Y., Milo T., Alon U. (2020). A new model for the HPA axis explains dysregulation of stress hormones on the timescale of weeks. Mol. Syst. Biol..

[bib90] Karpowicz P., Zhang Y., Hogenesch J.B., Emery P., Perrimon N. (2013). The circadian clock gates the intestinal stem cell regenerative state. Cell Rep.

[bib91] Kawamoto N., Ito H., Tokuda I.T., Iwasaki H. (2020). Damped circadian oscillation in the absence of KaiA in Synechococcus. Nat. Commun..

[bib92] Kim J.K., Chen Y., Hirning A.J., Alnahhas R.N., Josić K., Bennett M.R. (2019). Long-range temporal coordination of gene expression in synthetic microbial consortia. Nat. Chem. Biol..

[bib93] Kim J.K., Kilpatrick Z.P., Bennett M.R., Josić K. (2014). Molecular mechanisms that regulate the coupled period of the mammalian circadian clock. Biophys. J..

[bib94] Kim Y.-I., Dong G., Carruthers C.W., Golden S.S., LiWang A. (2008). The day/night switch in KaiC, a central oscillator component of the circadian clock of cyanobacteria. Proc. Natl. Acad. Sci. U. S. A..

[bib95] Kiss I.Z., Zhai Y., Hudson J.L. (2002). Emerging coherence in a population of chemical oscillators. Science.

[bib96] Ko C.H., Yamada Y.R., Welsh D.K., Buhr E.D., Liu A.C., Zhang E.E., Ralph M.R., Kay S.A., Forger D.B., Takahashi J.S. (2010). Emergence of noise-induced oscillations in the central circadian pacemaker. PLoS Biol.

[bib97] Kolbe I., Leinweber B., Brandenburger M., Oster H. (2019). Circadian clock network desynchrony promotes weight gain and alters glucose homeostasis in mice. Mol Metab.

[bib98] Kondo T., Strayer C.A., Kulkarni R.D., Taylor W., Ishiura M., Golden S.S., Johnson C.H. (1993). Circadian rhythms in prokaryotes: luciferase as a reporter of circadian gene expression in cyanobacteria. Proc. Natl. Acad. Sci. U. S. A..

[bib99] Konopka R.J., Benzer S. (1971). Clock mutants of Drosophila melanogaster. Proc. Natl. Acad. Sci. U. S. A..

[bib100] Korenčič A., Košir R., Bordyugov G., Lehmann R., Rozman D., Herzel H. (2014). Timing of circadian genes in mammalian tissues. Sci. Rep..

[bib101] Koronowski K.B., Kinouchi K., Welz P.-S., Smith J.G., Zinna V.M., Shi J., Samad M., Chen S., Magnan C.N., Kinchen J.M. (2019). Defining the Independence of the Liver Circadian Clock. Cell.

[bib102] Koronowski K.B., Sassone-Corsi P. (2021). Communicating clocks shape circadian homeostasis. Science.

[bib103] Kruse C.P.S., Meyers A.D., Basu P., Hutchinson S., Luesse D.R., Wyatt S.E. (2020). Spaceflight induces novel regulatory responses in Arabidopsis seedling as revealed by combined proteomic and transcriptomic analyses. BMC Plant Biol.

[bib104] Kume K., Zylka M.J., Sriram S., Shearman L.P., Weaver D.R., Jin X., Maywood E.S., Hastings M.H., Reppert S.M. (1999). mCRY1 and mCRY2 are essential components of the negative limb of the circadian clock feedback loop. Cell.

[bib105] Kunz H., Achermann P. (2003). Simulation of circadian rhythm generation in the suprachiasmatic nucleus with locally coupled self-sustained oscillators. J. Theor. Biol..

[bib106] Kuramoto Y. (1984). Cooperative dynamics of oscillator community: a study based on lattice of rings. Progr. Theoret. Phys..

[bib107] Kuramoto Y. (1975). International Symposium on Mathematical Problems in Theoretical Physics.

[bib108] Leise T.L., Wang C.W., Gitis P.J., Welsh D.K. (2012). Persistent cell-autonomous circadian oscillations in fibroblasts revealed by six-week single-cell imaging of PER2::LUC bioluminescence. PLoS One.

[bib109] Li G., Siddiqui H., Teng Y., Lin R., Wan X.-Y., Li J., Lau O.-S., Ouyang X., Dai M., Wan J. (2011). Coordinated transcriptional regulation underlying the circadian clock in Arabidopsis. Nat. Cell Biol..

[bib110] Li J.Z., Bunney B.G., Meng F., Hagenauer M.H., Walsh D.M., Vawter M.P., Evans S.J., Choudary P.V., Cartagena P., Barchas J.D. (2013). Circadian patterns of gene expression in the human brain and disruption in major depressive disorder. Proc. Natl. Acad. Sci. U. S. A..

[bib111] Li Q., Wang S., Milot E., Bergeron P., Ferrucci L., Fried L.P., Cohen A.A. (2015). Homeostatic dysregulation proceeds in parallel in multiple physiological systems. Aging Cell.

[bib112] Liu A.C., Welsh D.K., Ko C.H., Tran H.G., Zhang E.E., Priest A.A., Buhr E.D., Singer O., Meeker K., Verma I.M. (2007). Intercellular coupling confers robustness against mutations in the SCN circadian clock network. Cell.

[bib113] Liu C., Weaver D.R., Strogatz S.H., Reppert S.M. (1997). Cellular construction of a circadian clock: period determination in the suprachiasmatic nuclei. Cell.

[bib114] Li Y., Shan Y., Desai R.V., Cox K.H., Weinberger L.S., Takahashi J.S. (2020). Noise-driven cellular heterogeneity in circadian periodicity. Proc. Natl. Acad. Sci. U. S. A..

[bib115] Li Y., Shan Y., Kilaru G.K., Berto S., Wang G.-Z., Cox K.H., Yoo S.-H., Yang S., Konopka G., Takahashi J.S. (2020). Epigenetic inheritance of circadian period in clonal cells. eLife.

[bib116] Li Y., Wang L., Yuan L., Song Y., Sun J., Jia Q., Xie Q., Xu X. (2020). Molecular investigation of organ-autonomous expression of Arabidopsis circadian oscillators. Plant Cell Environ.

[bib117] Lundkvist G.B., Kwak Y., Davis E.K., Tei H., Block G.D. (2005). A calcium flux is required for circadian rhythm generation in mammalian pacemaker neurons. J. Neurosci..

[bib118] Lu S.X., Knowles S.M., Andronis C., Ong M.S., Tobin E.M. (2009). Circadian clock associated1 and late elongated hypocotyl function synergistically in the circadian clock of Arabidopsis. Plant Physiol.

[bib119] Lu S.X., Webb C.J., Knowles S.M., Kim S.H.J., Wang Z., Tobin E.M. (2012). CCA1 and ELF3 Interact in the control of hypocotyl length and flowering time in Arabidopsis. Plant Physiol.

[bib120] Marcheva B., Ramsey K.M., Buhr E.D., Kobayashi Y., Su H., Ko C.H., Ivanova G., Omura C., Mo S., Vitaterna M.H. (2010). Disruption of the clock components CLOCK and BMAL1 leads to hypoinsulinaemia and diabetes. Nature.

[bib121] Markson J.S., Piechura J.R., Puszynska A.M., O’Shea E.K. (2013). Circadian control of global gene expression by the cyanobacterial master regulator RpaA. Cell.

[bib122] Martins B.M., Das A., Antunes L., Locke J. (2016). Frequency doubling in the cyanobacterial circadian clock. Mol. Syst. Biol..

[bib123] Martin-Tryon E.L., Kreps J.A., Harmer S.L. (2007). GIGANTEA acts in blue light signaling and has biochemically separable roles in circadian clock and flowering time regulation. Plant Physiol.

[bib124] Maywood E.S., Chesham J.E., O’Brien J.A., Hastings M.H. (2011). A diversity of paracrine signals sustains molecular circadian cycling in suprachiasmatic nucleus circuits. Proc. Natl. Acad. Sci. U. S. A..

[bib125] Maywood E.S., Reddy A.B., Wong G.K.Y., O’Neill J.S., O’Brien J.A., McMahon D.G., Harmar A.J., Okamura H., Hastings M.H. (2006). Synchronization and maintenance of timekeeping in suprachiasmatic circadian clock cells by neuropeptidergic signaling. Curr. Biol..

[bib126] McMillen D., Kopell N., Hasty J., Collins J.J. (2002). Synchronizing genetic relaxation oscillators by intercell signaling. Proc. Natl. Acad. Sci. U. S. A..

[bib127] Medina F.-J., Herranz R. (2010). Microgravity environment uncouples cell growth and cell proliferation in root meristematic cells: the mediator role of auxin. Plant Signal. Behav..

[bib128] Michelet B., Chua N.-H. (1996). Improvement of arabidopsis mutant screens based on luciferase imaging in planta. Plant Mol. Biol. Rep..

[bib129] Mihalcescu I., Hsing W., Leibler S. (2004). Resilient circadian oscillator revealed in individual cyanobacteria. Nature.

[bib130] Millar A.J., Short S.R., Chua N.H., Kay S.A. (1992). A novel circadian phenotype based on firefly luciferase expression in transgenic plants. Plant Cell.

[bib131] Milo R. (2013). What is the total number of protein molecules per cell volume? A call to rethink some published values. Bioessays.

[bib132] Mizuno T., Nomoto Y., Oka H., Kitayama M., Takeuchi A., Tsubouchi M., Yamashino T. (2014). Ambient temperature signal feeds into the circadian clock transcriptional circuitry through the EC night-time repressor in Arabidopsis thaliana. Plant Cell Physiol.

[bib133] Mondragón-Palomino O., Danino T., Selimkhanov J., Tsimring L., Hasty J. (2011). Entrainment of a population of synthetic genetic oscillators. Science.

[bib134] Muranaka T., Oyama T. (2016). Heterogeneity of cellular circadian clocks in intact plants and its correction under light-dark cycles. Sci Adv.

[bib135] Myung J., Schmal C., Hong S., Tsukizawa Y., Rose P., Zhang Y., Holtzman M.J., De Schutter E., Herzel H., Bordyugov G., Takumi T. (2018). The choroid plexus is an important circadian clock component. Nat. Commun..

[bib136] Nagel D.H., Doherty C.J., Pruneda-Paz J.L., Schmitz R.J., Ecker J.R., Kay S.A. (2015). Genome-wide identification of CCA1 targets uncovers an expanded clock network in Arabidopsis. Proc. Natl. Acad. Sci. U. S. A..

[bib137] Nagoshi E., Saini C., Bauer C., Laroche T., Naef F., Schibler U. (2004). Circadian gene expression in individual fibroblasts: cell-autonomous and self-sustained oscillators pass time to daughter cells. Cell.

[bib138] Nakajima M., Imai K., Ito H., Nishiwaki T., Murayama Y., Iwasaki H., Oyama T., Kondo T. (2005). Reconstitution of circadian oscillation of cyanobacterial KaiC phosphorylation in vitro. Science.

[bib139] Nakamichi N., Ito S., Oyama T., Yamashino T., Kondo T., Mizuno T. (2004). Characterization of plant circadian rhythms by employing Arabidopsis cultured cells with bioluminescence reporters. Plant Cell Physiol..

[bib140] Nakamichi N., Kiba T., Henriques R., Mizuno T., Chua N.-H., Sakakibara H. (2010). Pseudo-Response Regulators 9, 7, and 5 are transcriptional repressors in the Arabidopsis circadian clock. Plant Cell.

[bib141] Nakamura T.J., Nakamura W., Tokuda I.T., Ishikawa T., Kudo T., Colwell C.S., Block G.D. (2015). Age-related changes in the circadian system unmasked by constant conditions. eNeuro.

[bib142] Niederholtmeyer H., Sun Z.Z., Hori Y., Yeung E., Verpoorte A., Murray R.M., Maerkl S.J. (2015). Rapid cell-free forward engineering of novel genetic ring oscillators. eLife.

[bib143] Nikhil K.L., Korge S., Kramer A. (2020). Heritable gene expression variability and stochasticity govern clonal heterogeneity in circadian period. PLoS Biol.

[bib144] Nimmo H.G. (2018). Entrainment of Arabidopsis roots to the light:dark cycle by light piping. Plant Cell Environ.

[bib145] Noguchi T., Wang L.L., Welsh D.K. (2013). Fibroblast PER2 circadian rhythmicity depends on cell density. J. Biol. Rhythms.

[bib146] Nohales M.A., Kay S.A. (2016). Molecular mechanisms at the core of the plant circadian oscillator. Nat. Struct. Mol. Biol..

[bib147] Nusinow D.A., Helfer A., Hamilton E.E., King J.J., Imaizumi T., Schultz T.F., Farré E.M., Kay S.A. (2011). The ELF4-ELF3-LUX complex links the circadian clock to diurnal control of hypocotyl growth. Nature.

[bib148] Omer Bendori S., Pollak S., Hizi D., Eldar A. (2015). The RapP-PhrP quorum-sensing system of Bacillus subtilis strain NCIB3610 affects biofilm formation through multiple targets, due to an atypical signal-insensitive allele of RapP. J. Bacteriol..

[bib149] O’Neill J.S., Maywood E.S., Chesham J.E., Takahashi J.S., Hastings M.H. (2008). cAMP-dependent signaling as a core component of the mammalian circadian pacemaker. Science.

[bib150] Ott E., Antonsen T.M. (2008). Low dimensional behavior of large systems of globally coupled oscillators. Chaos.

[bib151] Padovan-Merhar O., Nair G.P., Biaesch A.G., Mayer A., Scarfone S., Foley S.W., Wu A.R., Churchman L.S., Singh A., Raj A. (2015). Single mammalian cells compensate for differences in cellular volume and DNA copy number through independent global transcriptional mechanisms. Mol. Cell.

[bib152] Paijmans J., Bosman M., Ten Wolde P.R., Lubensky D.K. (2016). Discrete gene replication events drive coupling between the cell cycle and circadian clocks. Proc. Natl. Acad. Sci. U. S. A..

[bib153] Paijmans J., Lubensky D.K., Rein Ten Wolde P. (2017). Robustness of synthetic oscillators in growing and dividing cells. Phys Rev E.

[bib154] Palevitz B.A., Hepler P.K. (1985). Changes in dye coupling of stomatal cells of Allium and Commelina demonstrated by microinjection of Lucifer yellow. Planta.

[bib155] Pando M.P., Morse D., Cermakian N., Sassone-Corsi P. (2002). Phenotypic rescue of a peripheral clock genetic defect via SCN hierarchical dominance. Cell.

[bib156] Pattanayek R., Wang J., Mori T., Xu Y., Johnson C.H., Egli M. (2004). Visualizing a circadian clock protein: crystal structure of KaiC and functional insights. Mol. Cell.

[bib157] Paulmurugan R., Umezawa Y., Gambhir S.S. (2002). Noninvasive imaging of protein-protein interactions in living subjects by using reporter protein complementation and reconstitution strategies. Proc. Natl. Acad. Sci. U. S. A..

[bib158] Pett J.P., Korenčič A., Wesener F., Kramer A., Herzel H. (2016). Feedback Loops of the Mammalian Circadian Clock Constitute Repressilator. PLoS Comput. Biol..

[bib159] Pilorz V., Astiz M., Heinen K.O., Rawashdeh O., Oster H. (2020). The Concept of Coupling in the Mammalian Circadian Clock Network. J. Mol. Biol..

[bib160] Pokhilko A., Fernández A.P., Edwards K.D., Southern M.M., Halliday K.J., Millar A.J. (2012). The clock gene circuit in Arabidopsis includes a repressilator with additional feedback loops. Mol. Syst. Biol..

[bib161] Portolés S., Más P. (2010). The functional interplay between protein kinase CK2 and CCA1 transcriptional activity is essential for clock temperature compensation in Arabidopsis. PLoS Genet.

[bib162] Potvin-Trottier L., Lord N.D., Vinnicombe G., Paulsson J. (2016). Synchronous long-term oscillations in a synthetic gene circuit. Nature.

[bib163] Preitner N., Damiola F., Lopez-Molina L., Zakany J., Duboule D., Albrecht U., Schibler U. (2002). The orphan nuclear receptor REV-ERBα controls circadian transcription within the positive limb of the mammalian circadian oscillator. Cell.

[bib164] Prindle A., Samayoa P., Razinkov I., Danino T., Tsimring L.S., Hasty J. (2011). A sensing array of radically coupled genetic “biopixels”. Nature.

[bib165] Prindle A., Selimkhanov J., Li H., Razinkov I., Tsimring L.S., Hasty J. (2014). Rapid and tunable post-translational coupling of genetic circuits. Nature.

[bib166] Ralph M.R., Foster R.G., Davis F.C., Menaker M. (1990). Transplanted suprachiasmatic nucleus determines circadian period. Science.

[bib167] Ralph M.R., Menaker M. (1988). A mutation of the circadian system in golden hamsters. Science.

[bib168] Rascher U., Hütt M.T., Siebke K., Osmond B., Beck F., Lüttge U. (2001). Spatiotemporal variation of metabolism in a plant circadian rhythm: the biological clock as an assembly of coupled individual oscillators. Proc. Natl. Acad. Sci. U. S. A..

[bib169] Rawat R., Takahashi N., Hsu P.Y., Jones M.A., Schwartz J., Salemi M.R., Phinney B.S., Harmer S.L. (2011). REVEILLE8 and PSEUDO-REPONSE REGULATOR5 form a negative feedback loop within the Arabidopsis circadian clock. PLoS Genet.

[bib170] Rosario S.R., Long M.D., Affronti H.C., Rowsam A.M., Eng K.H., Smiraglia D.J. (2018). Pan-cancer analysis of transcriptional metabolic dysregulation using The Cancer Genome Atlas. Nat. Commun..

[bib171] Rosbash M. (2009). The implications of multiple circadian clock origins. PLoS Biol..

[bib172] Rosenfeld N., Young J.W., Alon U., Swain P.S., Elowitz M.B. (2005). Gene regulation at the single-cell level. Science.

[bib173] Rössler O.E. (1976). An equation for continuous chaos. Phys. Lett. A.

[bib174] Rougemont J., Naef F. (2007). Dynamical signatures of cellular fluctuations and oscillator stability in peripheral circadian clocks. Mol. Syst. Biol..

[bib175] Rugnone M.L., Faigón Soverna A., Sanchez S.E., Schlaen R.G., Hernando C.E., Seymour D.K., Mancini E., Chernomoretz A., Weigel D., Más P., Yanovsky M.J. (2013). LNK genes integrate light and clock signaling networks at the core of the Arabidopsis oscillator. Proc. Natl. Acad. Sci. U. S. A..

[bib176] Ruoff P., Rensing L. (1996). The temperature-compensated goodwin model simulates many circadian clock properties. J. Theor. Biol..

[bib177] Rust M.J., Markson J.S., Lane W.S., Fisher D.S., O’Shea E.K. (2007). Ordered phosphorylation governs oscillation of a three-protein circadian clock. Science.

[bib178] Saini C., Liani A., Curie T., Gos P., Kreppel F., Emmenegger Y., Bonacina L., Wolf J.-P., Poget Y.-A., Franken P., Schibler U. (2013). Real-time recording of circadian liver gene expression in freely moving mice reveals the phase-setting behavior of hepatocyte clocks. Genes Dev.

[bib179] Sakamoto K., Nagase T., Fukui H., Horikawa K., Okada T., Tanaka H., Sato K., Miyake Y., Ohara O., Kako K., Ishida N. (1998). Multitissue circadian expression of rat period homolog (rPer2) mRNA is governed by the mammalian circadian clock, the suprachiasmatic nucleus in the brain. J. Biol. Chem..

[bib180] Salas-González I., Reyt G., Flis P., Custódio V., Gopaulchan D., Bakhoum N., Dew T.P., Suresh K., Franke R.B., Dangl J.L. (2021). Coordination between microbiota and root endodermis supports plant mineral nutrient homeostasis. Science.

[bib181] Santos-Moreno J., Tasiudi E., Stelling J., Schaerli Y. (2020). Multistable and dynamic CRISPRi-based synthetic circuits. Nat. Commun..

[bib182] Schaffer R., Ramsay N., Samach A., Corden S., Putterill J., Carré I.A., Coupland G. (1998). The late elongated hypocotyl mutation of Arabidopsis disrupts circadian rhythms and the photoperiodic control of flowering. Cell.

[bib183] Schmal C., Herzog E.D., Herzel H. (2018). Measuring relative coupling strength in circadian systems. J. Biol. Rhythms.

[bib184] Schmal C., Myung J., Herzel H., Bordyugov G. (2017). Moran’s I quantifies spatio-temporal pattern formation in neural imaging data. Bioinformatics.

[bib185] Schmelling N.M., Lehmann R., Chaudhury P., Beck C., Albers S.-V., Axmann I.M., Wiegard A. (2017). Minimal tool set for a prokaryotic circadian clock. BMC Evol. Biol..

[bib186] Shearman L.P., Sriram S., Weaver D.R., Maywood E.S., Chaves I., Zheng B., Kume K., Lee C.C., van der Horst G.T., Hastings M.H., Reppert S.M. (2000). Interacting molecular loops in the mammalian circadian clock. Science.

[bib187] Siebke K., Weis E. (1995). Imaging of chlorophyll-a-fluorescence in leaves: Topography of photosynthetic oscillations in leaves of Glechoma hederacea. Photosynth. Res..

[bib188] Sinturel F., Gos P., Petrenko V., Hagedorn C., Kreppel F., Storch K.-F., Knutti D., Liani A., Weitz C., Emmenegger Y. (2021). Circadian hepatocyte clocks keep synchrony in the absence of a master pacemaker in the suprachiasmatic nucleus or other extrahepatic clocks. Genes Dev.

[bib189] Stephan F.K., Zucker I. (1972). Circadian rhythms in drinking behavior and locomotor activity of rats are eliminated by hypothalamic lesions. Proc. Natl. Acad. Sci. U. S. A..

[bib190] St John P.C., Doyle F.J. (2015). Quantifying Stochastic Noise in Cultured Circadian Reporter Cells. PLoS Comput. Biol..

[bib191] St John P.C., Hirota T., Kay S.A., Doyle F.J. (2014). Spatiotemporal separation of PER and CRY posttranslational regulation in the mammalian circadian clock. Proc. Natl. Acad. Sci. U. S. A..

[bib192] Stokes K., Cooke A., Chang H., Weaver D.R., Breault D.T., Karpowicz P. (2017). The circadian clock gene BMAL1 coordinates intestinal regeneration. Cell. Mol. Gastroenterol. Hepatol..

[bib193] Stokkan K.A., Yamazaki S., Tei H., Sakaki Y., Menaker M. (2001). Entrainment of the circadian clock in the liver by feeding. Science.

[bib194] Stricker J., Cookson S., Bennett M.R., Mather W.H., Tsimring L.S., Hasty J. (2008). A fast, robust and tunable synthetic gene oscillator. Nature.

[bib195] Strogatz S.H. (2000). From Kuramoto to Crawford: exploring the onset of synchronization in populations of coupled oscillators. Physica D.

[bib196] Süel G.M., Kulkarni R.P., Dworkin J., Garcia-Ojalvo J., Elowitz M.B. (2007). Tunability and noise dependence in differentiation dynamics. Science.

[bib197] Tahara Y., Kuroda H., Saito K., Nakajima Y., Kubo Y., Ohnishi N., Seo Y., Otsuka M., Fuse Y., Ohura Y. (2012). In vivo monitoring of peripheral circadian clocks in the mouse. Curr. Biol..

[bib198] Takahashi N., Hirata Y., Aihara K., Mas P. (2015). A hierarchical multi-oscillator network orchestrates the Arabidopsis circadian system. Cell.

[bib199] Taylor S.R., Wang T.J., Granados-Fuentes D., Herzog E.D. (2017). Resynchronization dynamics reveal that the ventral entrains the dorsal suprachiasmatic nucleus. J. Biol. Rhythms.

[bib200] Thain S.C., Hall A., Millar A.J. (2000). Functional independence of circadian clocks that regulate plant gene expression. Curr. Biol..

[bib201] Thain S.C., Murtas G., Lynn J.R., McGrath R.B., Millar A.J. (2002). The circadian clock that controls gene expression in Arabidopsis is tissue specific. Plant Physiol.

[bib202] Tigges M., Dénervaud N., Greber D., Stelling J., Fussenegger M. (2010). A synthetic low-frequency mammalian oscillator. Nucleic Acids Res.

[bib203] Tigges M., Marquez-Lago T.T., Stelling J., Fussenegger M. (2009). A tunable synthetic mammalian oscillator. Nature.

[bib204] To T.-L., Henson M.A., Herzog E.D., Doyle F.J. (2007). A molecular model for intercellular synchronization in the mammalian circadian clock. Biophys. J..

[bib205] Triqueneaux G., Thenot S., Kakizawa T., Antoch M.P., Safi R., Takahashi J.S., Delaunay F., Laudet V. (2004). The orphan receptor Rev-erbα gene is a target of the circadian clock pacemaker. J. Mol. Endocrinol..

[bib206] Tsai T.Y.-C., Choi Y.S., Ma W., Pomerening J.R., Tang C., Ferrell J.E. (2008). Robust, tunable biological oscillations from interlinked positive and negative feedback loops. Science.

[bib207] Ueda H.R., Hayashi S., Chen W., Sano M., Machida M., Shigeyoshi Y., Iino M., Hashimoto S. (2005). System-level identification of transcriptional circuits underlying mammalian circadian clocks. Nat. Genet..

[bib208] Ukai H., Ueda H.R. (2010). Systems biology of mammalian circadian clocks. Annu. Rev. Physiol..

[bib209] Ukai K., Murase H., Fukuda H. (2013). Spatiotemporal dynamics of circadian clock in lettuce. IFAC Proc. Vol..

[bib210] Ukai-Tadenuma M., Yamada R.G., Xu H., Ripperger J.A., Liu A.C., Ueda H.R. (2011). Delay in feedback repression by Cryptochrome 1 is required for circadian clock function. Cell.

[bib211] Uzumaki T., Fujita M., Nakatsu T., Hayashi F., Shibata H., Itoh N., Kato H., Ishiura M. (2004). Crystal structure of the C-terminal clock-oscillator domain of the cyanobacterial KaiA protein. Nat. Struct. Mol. Biol..

[bib212] Walker N., Nghe P., Tans S.J. (2016). Generation and filtering of gene expression noise by the bacterial cell cycle. BMC Biol.

[bib213] Wang Y., Wu J.-F., Nakamichi N., Sakakibara H., Nam H.-G., Wu S.-H. (2011). LIGHT-REGULATED WD1 and PSEUDO-RESPONSE REGULATOR9 form a positive feedback regulatory loop in the Arabidopsis circadian clock. Plant Cell.

[bib214] Wang Z.Y., Tobin E.M. (1998). Constitutive expression of the CIRCADIAN CLOCK ASSOCIATED 1 (CCA1) gene disrupts circadian rhythms and suppresses its own expression. Cell.

[bib215] Webb A.B., Angelo N., Huettner J.E., Herzog E.D. (2009). Intrinsic, nondeterministic circadian rhythm generation in identified mammalian neurons. Proc. Natl. Acad. Sci. U. S. A..

[bib216] Webb A.B., Taylor S.R., Thoroughman K.A., Doyle F.J., Herzog E.D. (2012). Weakly circadian cells improve resynchrony. PLoS Comput. Biol..

[bib217] Welsh D.K., Logothetis D.E., Meister M., Reppert S.M. (1995). Individual neurons dissociated from rat suprachiasmatic nucleus express independently phased circadian firing rhythms. Neuron.

[bib218] Welsh D.K., Yoo S.-H., Liu A.C., Takahashi J.S., Kay S.A. (2004). Bioluminescence imaging of individual fibroblasts reveals persistent, independently phased circadian rhythms of clock gene expression. Curr. Biol..

[bib219] Wenden B., Toner D.L.K., Hodge S.K., Grima R., Millar A.J. (2012). Spontaneous spatiotemporal waves of gene expression from biological clocks in the leaf. Proc. Natl. Acad. Sci. U. S. A..

[bib220] Westermark P.O., Welsh D.K., Okamura H., Herzel H. (2009). Quantification of circadian rhythms in single cells. PLoS Comput. Biol..

[bib221] Winfree A.T. (1967). Biological rhythms and the behavior of populations of coupled oscillators. J. Theor. Biol..

[bib222] Wu J.-F., Tsai H.-L., Joanito I., Wu Y.-C., Chang C.-W., Li Y.-H., Wang Y., Hong J.C., Chu J.-W., Hsu C.-P., Wu S.-H. (2016). LWD-TCP complex activates the morning gene CCA1 in Arabidopsis. Nat. Commun..

[bib223] Wu L., Ouyang Q., Wang H. (2017). Robust network topologies for generating oscillations with temperature-independent periods. PLoS ONE.

[bib224] Xie Q., Wang P., Liu X., Yuan L., Wang L., Zhang C., Li Y., Xing H., Zhi L., Yue Z. (2014). LNK1 and LNK2 are transcriptional coactivators in the Arabidopsis circadian oscillator. Plant Cell.

[bib225] Yakir E., Hassidim M., Melamed-Book N., Hilman D., Kron I., Green R.M. (2011). Cell autonomous and cell-type specific circadian rhythms in Arabidopsis: Circadian rhythms in individual plant cells. Plant J.

[bib226] Yakir E., Hilman D., Kron I., Hassidim M., Melamed-Book N., Green R.M. (2009). Posttranslational regulation of CIRCADIAN CLOCK ASSOCIATED1 in the circadian oscillator of Arabidopsis. Plant Physiol.

[bib227] Yamaguchi S., Isejima H., Matsuo T., Okura R., Yagita K., Kobayashi M., Okamura H. (2003). Synchronization of cellular clocks in the suprachiasmatic nucleus. Science.

[bib228] Yamaguchi S., Mitsui S., Miyake S., Yan L., Onishi H., Yagita K., Suzuki M., Shibata S., Kobayashi M., Okamura H. (2000). The 5′ upstream region of mPer1 gene contains two promoters and is responsible for circadian oscillation. Curr. Biol..

[bib229] Yamazaki S., Numano R., Abe M., Hida A., Takahashi R., Ueda M., Block G.D., Sakaki Y., Menaker M., Tei H. (2000). Resetting central and peripheral circadian oscillators in transgenic rats. Science.

[bib230] Yamazaki S., Straume M., Tei H., Sakaki Y., Menaker M., Block G.D. (2002). Effects of aging on central and peripheral mammalian clocks. Proc. Natl. Acad. Sci. U. S. A..

[bib231] Yoo S.-H., Yamazaki S., Lowrey P.L., Shimomura K., Ko C.H., Buhr E.D., Siepka S.M., Hong H.-K., Oh W.J., Yoo O.J. (2004). PERIOD2::LUCIFERASE real-time reporting of circadian dynamics reveals persistent circadian oscillations in mouse peripheral tissues. Proc. Natl. Acad. Sci. U. S. A..

[bib232] Yoshitane H., Asano Y., Sagami A., Sakai S., Suzuki Y., Okamura H., Iwasaki W., Ozaki H., Fukada Y. (2019). Functional D-box sequences reset the circadian clock and drive mRNA rhythms. Commun Biol.

[bib233] Zehring W.A., Wheeler D.A., Reddy P., Konopka R.J., Kyriacou C.P., Rosbash M., Hall J.C. (1984). P-element transformation with period locus DNA restores rhythmicity to mutant, arrhythmic Drosophila melanogaster. Cell.

[bib234] Zhang E.E., Liu A.C., Hirota T., Miraglia L.J., Welch G., Pongsawakul P.Y., Liu X., Atwood A., Huss J.W., Janes J. (2009). A genome-wide RNAi screen for modifiers of the circadian clock in human cells. Cell.

